# The Contributions of Multiple Polygenic Scores in Predicting Liability for Major Depressive Disorder and Its Comorbidity with Alcohol Use Disorder

**DOI:** 10.1007/s10519-026-10263-3

**Published:** 2026-04-10

**Authors:** Jonathan L. Wells, Jill A. Rabinowitz, Brion S. Maher, Amanda E. Gentry, James B. Burch, Elizabeth C. Prom-Wormley

**Affiliations:** 1 Department of Epidemiology, Virginia Commonwealth University School of Public Health, 830 E Main St. Floor 8, Richmond, VA 23219 USA; 2 Virginia Institute for Psychiatric and Behavioral Genetics, Virginia Commonwealth University, Richmond, VA 23298 USA; 3 Department of Psychiatry, Robert Wood Johnson Medical School, Rutgers University, New Brunswick, NJ 08901 USA; 4 Department of Mental Health, Bloomberg School of Public Health, Johns Hopkins University, Baltimore, MD 21205 USA; 5 BeOne Medicines Ltd, Basel, Switzerland

**Keywords:** Major depressive disorder, Alcohol use disorder, Comorbidity, Polygenic scores, Genetic liability, Ancestry

## Abstract

The inclusion of polygenic scores (PGS) from genetically correlated traits such as Major Depressive Disorder (MDD) and alcohol use disorder (AUD) may improve the prediction of these outcomes and their comorbidity. Despite the importance of this work, few studies have evaluated the efficacy of this possibility. The current study evaluates the use of MDD and AUD PGS individually and together to improve the prediction of MDD, AUD, and comorbid MDD-AUD using a sample of European, African, or Admixed American Ancestry participants from the National Longitudinal Study of Adolescent to Adult Health (*N* = 7,965). Cross-ancestry MDD and AUD PGS were created using PRS-CSx. The best fitting model of comorbid MDD-AUD in the whole sample included PGS for MDD and AUD (PGS_MDD_ OR: 1.26, 95% CI 1.16–1.35, *p* = 2.69 × 10^− 6^; PGS_AUD_ OR: 1.77, 95% CI 1.66–1.87, *p* = 3.49 × 10^− 28^), explaining an additional 4.88% of variance compared to a model only including sociodemographic covariates. For MDD, the best fitting model included the MDD PGS (OR: 1.25, 95% CI 1.17–1.33, *p* = 2.05 × 10^− 8^), explaining an additional 0.65% of variance. For AUD, the best fitting model included the AUD PGS (OR: 1.37, 95% CI 1.32–1.43, *p* = 1.25 × 10^−28^), which explained an additional 1.52% of variance. Inclusion of both PGS did not significantly improve the prediction of individual MDD or AUD. Inclusion of PGS for MDD and AUD significantly improved prediction for comorbid MDD-AUD, but not in MDD or AUD. These results help clarify the role of utilizing genetically correlated PGS in improving prediction of MDD, AUD, and comorbid MDD-AUD.

**Supplementary Information:**

The online version contains supplementary material available at 10.1007/s10519-026-10263-3.

## Introduction

Major depressive disorder (MDD, i.e., mood disorder characterized by symptoms of low mood, loss of interest, and feelings of excessive guilt) and AUD (i.e., challenges with controlling drinking habits and alcohol consumption, despite adverse social or health consequences) each represent some of the most common mental health and substance use outcomes in the United States (American Psychiatric Association 2013). Approximately 20.6% and 29.1% of individuals in the U.S. were estimated to have lifetime MDD and AUD, in 2018 and 2015, respectively (Grant et al. 2015; Hasin et al. 2018).

In clinical settings, comorbidity is commonly defined as the presence of two or more concurrent distinct medical conditions, constituting the need for distinct additional clinical entities in the context of the timing and affected anatomic locations when considering a comorbid diagnosis (Feinstein 1970; Valderas et al. 2009). However, the use of this term in psychiatric and substance use disorders requires more nuance (Nordgaard et al. 2023). Considering the overlapping symptoms, the persistent nature, and unclear etiology of these disorders, the field has often focused on a definition of psychiatric comorbidity that observes co-occurrence of the disorders across the lifespan (Maj 2005; First 2005; Kessler et al. 2012; Nordgaard et al. 2023).

Using this definition, it has been observed that many individuals affected by MDD or AUD are often also affected with the other condition as well. In a longitudinal study it was estimated that approximately 40% of lifetime MDD patients had a history of AUD, similarly, 59% of lifetime AUD patients had a history of MDD (Brière et al. 2014). Patients affected with comorbid MDD with AUD experience more severe symptoms and outcomes compared to those with either MDD or AUD alone (Brière et al. 2014; Conner et al. 2014; McHugh and Weiss 2019). Compared to patients with either MDD or AUD only, patients with comorbid MDD-AUD experience symptoms that are more difficult to treat (Davis et al. 2008, 2010), as evidenced by higher rates of alcohol dependence, AUD relapse (Hasin et al. 2002), and treatment-resistant MDD (Blanco et al. 2012). Therefore, comorbid MDD-AUD represents a substantial burden to the U.S. healthcare system as well as social support and clinical care systems that support individuals affected with these conditions (Davis et al. 2008, 2010; Rehm et al. 2009; Greenberg et al. 2021).These conditions are often studied independently, limiting the ability to effectively address the shared symptoms, psychosocial risk factors, and common genetic effects contributing to their comorbidity (Bourque et al. 2024).

### Genetic Factors Contributing to MDD May Also Influence AUD

Twin studies and genome-wide association studies (GWAS) point towards a significant contribution of common genetic effects on MDD and AUD. MDD and AUD are moderately heritable traits with twin studies reporting a 31–42% heritability for MDD and 43–53% heritability for AUD, with the remainder of the phenotypic variance being attributed to the specific environment of individuals (Sullivan et al. 2000; Verhulst et al. 2015). Additionally, the most recent MDD GWAS reported significant associations with 243 independent genetic loci, resulting in SNP-based heritability estimates ranging from 8.9% to 16.7%. (Howard et al. 2018; Wray et al. 2018; Als et al. 2022; Meng et al. 2024). To date, GWAS investigating problematic alcohol use, including AUD, reported associations at 110 independent genetic loci, with an estimated SNP-based heritability ranging from 5.6% to 16.2% (Sanchez-Roige et al. 2019; Kranzler et al. 2019; Zhou et al. 2020, 2023). Further, many of the genetic factors contributing to MDD are also likely to contribute to AUD, with genetic correlation estimates ranging between 0.52 and 0.63 in GWAS and twin studies (Kendler et al. 1993, 1997; Prescott et al. 2000; Ellingson et al. 2016; Walters et al. 2018).

The high rates of comorbidity and moderate genetic correlations suggest that the genetic liabilities for MDD and AUD are not independent and may work together to influence liability for comorbid MDD-AUD, i.e., through pleiotropy (Sivakumaran et al. 2011; Chesmore et al. 2018; Chebib and Guillaume 2021). Pleiotropy occurs when a genetic variant is associated with more than one trait (Sivakumaran et al. 2011; Chesmore et al. 2018; Chebib and Guillaume 2021); pleiotropic associations are ubiquitous across the genome and can arise from: (Albiñana et al. 2022) horizonal pleiotropy, where a single variant is directly implicated in both traits; (Als et al. 2022) vertical pleiotropy, where a single variant is implicated in one trait that is along the causal pathway to another trait; (Altshuler et al. 2010) and spurious pleiotropy, where the variant is only associated with one trait and mistakenly associated with another trait (Chesmore et al. 2018). To date, genomic research on the comorbidity of MDD and AUD has not yet offered clarity on the nature of the pleiotropy between these outcomes (Jee et al. 2025). This aligns with ambiguity in epidemiologic results, where comorbid patients do not follow a consistent pattern of disorder diagnosis progression (Brière et al. 2014). To address this gap, this study considers the role of multiple genetic variants that may contribute to either the horizontal or vertical pleiotropy underlying comorbid MDD-AUD as well as their contribution to each individual outcome.

### Inclusion of Polygenic Scores for AUD and MDD May Improve Classification for Both Conditions and Their Comorbidity

Well-powered and generalizable genome-wide polygenic scores (PGS, an index of common genetic effects that contribute to a trait across all measured loci in the genome) for MDD and AUD are expected to have multiple clinical implications, including: (Albiñana et al. 2022) providing clinicians with more information during the decision-making process for treatment plans (Als et al. 2022), the identification of MDD and AUD patients who have a higher odds of developing comorbid MDD-AUD, and (Altshuler et al. 2010) the identification of higher genetic risk individuals to enroll in preventative public health programs (Palk et al. 2019; Barr et al. 2020). To date, PGS for MDD and AUD have been estimated but only explain a small proportion of the total variance of each outcome. Prior studies report that MDD PGS explain 0.8–3.2% of the phenotypic variance for MDD (Howard et al. 2018; Navrady et al. 2018; Rabinowitz et al. 2020; Mitchell et al. 2021; Meng et al. 2024). Further, AUD PGS explain 0.1%-3.5% for AUD (Walters et al. 2018; Zhou et al. 2020, 2023). MDD and AUD PGS are subject to some limitations that have impeded their clinically implementation (Andersen et al. 2017; Palk et al. 2019; Barr et al. 2020).

First, there is the concern of low statistical power in the original discovery GWAS used to generate the PGS, which can be improved by increasing the sample size or increasing the specificity of the outcome measure. Another concern is the generalizability of the PGS, most PGS have been generated using discovery GWAS of primarily European descent, which are ineffective in non-European descent populations (Wang et al. 2023). To counteract this, more efforts are being made to increase the sample size of non-European GWAS, and methods have been created to investigate and utilize cross ancestry and ancestry specific patterns of common genetic variance (Ruan et al. 2022; Zhou et al. 2023; Meng et al. 2024). However, efforts to utilize these data and methods to generate improved PGS for MDD and AUD are still lagging, thus it is important to utilize this data and methods to investigate cross-ancestry and ancestry-specific PGS effects for MDD and AUD, which was done in the present study.

An additional issue in genetic research of MDD, AUD, and comorbid MDD-AUD is the diagnosis of these conditions are subject to large amounts of phenotypic heterogeneity (Torvik et al. 2017; Wendt et al. 2020). For example, in MDD, there are a possible 227 unique symptom presentations that would be diagnosed as MDD under the DSM-5 definition. Further, many studies have presented strong evidence for clustering MDD patients into subtypes to inform treatment based on biological and behavioral factors (Østergaard et al. 2011; Tozzi et al. 2024; Tang et al. 2025). Similarly, for AUD, the DSM-5 definition allows for multiple levels of severity and significant research has been directed towards isolating clinical and biological subtypes associated with symptom presentation and alcohol use behaviors (Müller et al. 2020; Zhu et al. 2022). Further, comorbid MDD-AUD patients are different in symptom presentation and treatment resistance from either MDD or AUD patients, supporting the concept that the comorbid condition could be associated with specific subtypes of the single conditions (Hasin et al. 2002; Blanco et al. 2012; Brière et al. 2014; McHugh and Weiss 2019). Currently, genetic studies of MDD and AUD do not fully consider this heterogeneity in their study designs, limiting the predictive ability of the PGS. However, it is possible to address a portion of this heterogeneity in current genetic studies, specifically by accounting for the shared genetic effects between the disorders and how they may relate to the comorbid disorder.

Despite the observed heterogeneity and shared genetic effects in MDD, AUD, and the comorbid condition, very few studies have investigated the distinct effects of AUD or MDD PGS on the other respective disorder, and no studies have investigated these effects in the comorbid condition. It follows that the incorporation of genetic effects that contribute to MDD as well as AUD may improve the utility of PGS for either condition or comorbid MDD-AUD. Some studies use PGS of multiple traits—guided by genetic correlation estimates—to improve prediction (Jansen et al. 2021; Albiñana et al. 2022); these studies demonstrate the importance of utilizing shared genetic effects for single disorder prediction, but do not evaluate the scores with respect to comorbid condition prediction. Further, inclusion of MDD PGS has improved the prediction of alcohol dependence and alcohol consumption (Andersen et al. 2017; Karpyak et al. 2023). The extent to which the study of MDD, AUD, and comorbid MDD-AUD would benefit from the use of MDD and AUD PGS together remains unclear, as few studies have investigated these effects.

To address the aforementioned limitations, this study uses data from a U.S. nationally representative cohort of individuals assessed from adolescence through adulthood to estimate the ability of MDD and AUD PGS individually and jointly to predict MDD, AUD, and comorbid MDD-AUD. Specifically, this study explores the degree to which the AUD and MDD PGS are associated with AUD, MDD, and comorbid MDD-AUD, when used singularly and when used together. Additionally, this study evaluates the degree to which the amount of phenotypic variance explained by PGS for MDD and AUD differs between models of MDD, AUD, and comorbid MDD-AUD.

## Methods

### Discovery Samples

Summary statistics for MDD and AUD from two different discovery samples were used as described below. The MDD and AUD GWAS sample sizes for each ancestry group in these samples are summarized in Table 1.

**Table 1:** Discovery GWAS case and control sample size by ancestry

Ancestry group	MDD cases	MDD control	AUD cases	AUD control
EUR	246,241	558,568	112,557	636,942
AFR	36,818	161,679	40,116	82,455
AMR	25,013	352,946	10,150	28,812

*EUR* European Ancestry, *AFR* African Ancestry, *AMR* Ad-mixed American Ancestry

#### Major Depressive Disorder

MDD analyses used ancestry-specific genotypic summary statistics produced from a consortium-based GWAS (Meng et al. 2024). This study included data from the following studies: PGC-MDD2, Intern Health Study, UK Biobank, Army-STARRS, BioMe, Million Veterans Program, Genetic Epidemiology Research on Aging, Drakenstien Child Health Study, Vanderbilt University Biobank, Detroit Neighborhood Health Study, Prevention Intervention Research Center, Hispanic Community Health Study, Mexican Adolescent Mental Health Survey, Pregnancy Outcomes Maternal and Infant Study. This discovery sample represents the largest trans-ancestry GWAS of MDD to date.

MDD case status was defined as a binary variable where individuals affected with MDD were coded as “1” and non-affected individuals were coded as “0”. This study used a broad definition to determine MDD status that was designed to incorporate multiple methods of MDD measurement to increase sample size. These methods of MDD measurement included structured interviews for DSM-IV, DSM-5, ICD 9, and ICD 10 MDD criterium, medical record indicators (diagnosis or prescription of antidepressants), or a self-reported diagnosis of MDD (Meng et al. 2024). Despite differences in how MDD was assessed (e.g., diagnostic interviews, self-reports), there is a substantial phenotypic and genetic correlation across measures. For example, the correlations between the gold standard structural interviews for DSM-5 and self-reported MDD are between 0.60 and 0.90 (Sanchez-Villegas et al. 2008; Stuart et al. 2014; Davies et al. 2022), while the genetic correlations of all the MDD measures included in the MDD GWAS were between 0.85 and 0.89 (Meng et al. 2024). For the MDD discovery sample in this analysis, three ancestry-specific summary statistics were used: European Ancestry (EUR), African Ancestry (AFR), and Admixed American Ancestry (AMR).

#### Alcohol Use Disorder

AUD summary statistic results from a recent trans-ancestry problematic alcohol use GWAS were used for this analysis (Zhou et al. 2023). The previous study meta-analyzed results across included individuals from the following studies: PGC-AD, Million Veterans Program, FinnGen, UK Biobank, Queensland Institute of Medical Research, iPSYCH, Yale-Penn 3, Han Chinese–Illumina Global Screening Array, Han Chinese–Illumina Cyto12 array, Thai METH. This GWAS represents the largest trans-ancestry GWAS of AUD to date.

In the AUD GWAS, AUD was assessed across several measures of alcohol related disorders, including: (Albiñana et al. 2022) structured interviews of AUD using the DSM-IV and DSM-5 (Als et al. 2022), ICD 9 and ICD-10 diagnosis of AUD and (Altshuler et al. 2010) the DSM-IV diagnosis of alcohol dependence. Small phenotypic and genotypic differences have been found between DSM-IV/DSM-5/ICD-9/ICD-10 definitions of AUD or DSM-IV definitions of AD with genetic correlations around 0.98 (Dawson et al. 2013; Takahashi et al. 2017; Zhou et al. 2020).

AUD summary statistics were drawn from a subset of participants with an AUD diagnosis and who did not participate in the target sample. This decision was taken because the DSM-IV definition of AUD was used in the target dataset for subsequent analyses. Initially, the discovery sample included many participants measured with the Alcohol Use Disorders Identification Test problems subscale scores (AUDIT-P), which were not included in the summary statistics used for this analysis. Inclusion of the AUDIT-P summary statistics in the PGS generation could have induced bias into the PGS estimates of the target sample, since the overlap between the genetic factors contributing to AUD and the AUDIT-P was not as strong (r_g_ = 0.71) (Zhou et al. 2023). Additionally, the target sample for this study was initially included in the EUR AUD discovery sample, thus these participants were removed from the summary statistics used for this analysis, to avoid inflation of results due to overlap between discovery and target samples. Three ancestry-specific summary statistics from the AUD discovery sample were applied in the target sample: EUR, AFR, and AMR.

### Target Sample

Data from the National Longitudinal Study of Adolescent to Adult Health (Add Health) were used as the target sample to evaluate the PGS created based on the discovery samples described above (Harris et al. 2019). Add Health is a large nationally representative longitudinal study that began in 1994. Initially, over 90,000 high school students from 144 schools across the U.S. were recruited for an in-school study. From this initial study, a large portion of students were recruited using complex survey sampling methods, recruiting 20,745 students aged 12–19 years old at the first timepoint (Wave I; W1) in 1994–1995. These participants were contacted for follow-up an additional four times at Wave II (W2;1996), Wave III (W3; 2001–2002), Wave IV (W4; 2008–2009), and Wave V (W5; 2016–2018). All participants responded to questions about adolescent social, psychological, and biological factors during Waves I-III. They also responded to questions about adulthood social, psychological, and biological outcomes at Waves III-V (Harris et al. 2019). Participants provided a saliva sample for genetic analyses at W1 but were asked for consent to use their genetic data at W4. All participants in W4 were also in W1.

Analyses were limited to W4 participants who consented to the use of their genetic data in the dbGAP repository, passed genetic quality control measures (discussed below), and had measurements for MDD and AUD case status at W4. Of the 15,701 W4 participants, 12,234 consented to the use of their genetic data in dbGaP, 9974 of which passed quality control measures. There is a significant number of twins and siblings (*n* = 1414, 16.3%) in the Add Health sample. To avoid bias in the PGS estimates caused by shared genetic and environmental effects in sibling relationships, only one member of each family was included in this analysis (*n* = 693). When comparing these individuals to the rest of the population, no significant differences were found in outcome prevalence or PGS, indicating minimal selection bias caused by the oversampling of siblings in this analysis (Supplemental Table 1). Analyses were conducted among all remaining participants who were genetically similar to European Ancestry (EUR; *N* = 5218), African Ancestry (AFR; *N* = 1836), Admixed American Ancestry (AMR; *N* = 911). Participants were determined to be EUR or AMR ancestry if they fell within one standard deviation of the respective self-reported non-Hispanic White or Hispanic race/ethnicity group means of the first and second ancestry principal components (Braudt and Harris 2020). Participants were categorized as AFR if they self-identified as non-Hispanic Black race/ethnicity and had a value for the first principal component that was within two standard deviations of the non-Hispanic Black group mean of the first ancestry principal component and within one standard deviation of the non-Hispanic Black group mean of the second ancestry principal component (Braudt and Harris 2020). Due to the large amount of relatedness within the Add Health sample the genetic ancestry of all unrelated participants was determined by the Add Health study through the use of GRAF-pop, and then the estimates were then projected onto the remaining related participants (Jin et al. 2019).

### Outcome Definitions

Lifetime MDD was defined using a self-reported item at W4, “Have you ever been diagnosed with depression?”. Participants who responded “yes” were considered to be affected with MDD and were coded as “1”. Those who responded “no” were considered to not be affected and were coded as “0”. Participants who indicated having lifetime AUD were excluded from the MDD analyses. Over half of the participants included in the broad MDD definition were of a similar self-reported measure (Sanchez-Villegas et al. 2008; Stuart et al. 2014; Davies et al. 2022; Meng et al. 2024). Therefore, the MDD definitions in the target and discovery samples are expected to be well aligned for this analysis.

Lifetime AUD was defined as being affected by lifetime DSM-IV AUD at W4, which is derived through indication of either alcohol dependence or alcohol abuse. This definition has a large amount of concordance with the current DSM-5 measure of AUD (Dawson et al. 2013; Takahashi et al. 2017; Bailey et al. 2025). Participants responded to a DSM-IV AUD checklist that included seven questions related to alcohol dependence symptoms and four to alcohol abuse symptoms (Supplemental Table 2). These checklist questions were asked to participants who had indicated they consumed more than 2–3 drinks of alcohol in their lifetime. Participants were coded as being affected with AUD (coded as “1”) if they responded “Yes” to at least three of the seven alcohol dependence questions (indicating alcohol dependence) or responded “more than once” to at least one of the four alcohol abuse questions (indicating alcohol abuse). Participants were coded as “0” otherwise, including if they answered “No” to ever consuming more than two or three drinks of alcohol in their lifetime. Participants who indicated having lifetime MDD were excluded from the AUD analyses. DSM-IV definitions of AUD were included as part of the discovery sample definition of AUD. Genetic correlations between AUD definitions were estimated to be 0.98 (Zhou et al. 2023). This suggests that the target and discovery data definitions of AUD should be well aligned for this analysis.

Lifetime comorbid MDD-AUD was defined as a positive indication of both lifetime MDD and AUD at W4. Specifically, those with a “1” for both MDD and AUD were a “1” for comorbid MDD-AUD, those with “0” for both MDD and AUD were a “0” for comorbid MDD-AUD, and those with just one diagnosis were excluded from comorbid MDD-AUD analyses. This definition does not specifically account for the concurrent diagnosis of MDD and AUD, but the occurrence of lifetime MDD and AUD in the same individual at any point in their life leading up to the time they were interviewed.

### Covariates

The contribution of several additional variables were included in each model to account for their contributions to the associations between the PGS with MDD, AUD, and comorbid MDD-AUD.

#### Age

Age is an important factor in MDD and AUD. The highest prevalence of both occurs between the ages of 18 and 29 (Seeley et al. 2019; Villarroel and Terlizzi 2020). Data collection for MDD and AUD as well as age occurred during W4. Age at W4 interview was treated as a continuous variable.

#### Race/Ethnicity

The prevalence of MDD and AUD consistently differs between racial and ethnic groups (Yoon et al. 2015; Villarroel and Terlizzi 2020; Substance Abuse and Mental Health Services Administration 2022). Self-reported race/ethnicity at W1 was originally measured as a 5-level categorical variable: White, Black, Hispanic, Asian, and Other.

#### Sex

Lifetime MDD prevalence in females is almost twice that of males, while lifetime AUD prevalence in males is close to double that of females (Cerdá et al. 2011; Brière et al. 2014; Collins 2016; Hasin et al. 2018; Substance Abuse and Mental Health Services Administration 2022; Zare et al. 2022; Benny et al. 2023). Biological sex was determined by Add Health by checking the heterozygosity rate of the X chromosome of individuals and validating it against their self-reported gender. Individuals whose biological sex and self-reported gender did not match were excluded from this analysis. Biological sex was treated as a binary variable (Male/Female).

#### Adolescent and Adulthood Income

Lower levels of parental household income during adolescence and adulthood household income are consistently associated with higher risk for MDD and AUD (Cerdá et al. 2011; Collins 2016; Zare et al. 2022; Benny et al. 2023). Parental household income during adolescence at W1 and household income during adulthood at W4 were included as covariates. Both income variables were treated as continuous variables after first being categorized using the following eight levels: (1) less than $10,000 (2), $10,000 to $19,999 (3), $20,000 to $29,999 (4), $30,000 to $39,999 (5), $40,000 to $49,999 (6), $50,000 to $74,999 (7), $75,000 to $99,999, and (8) $100,000 or more.

#### Educational Attainment

Lower adulthood educational attainment has been linked with increased risk for MDD and AUD (Lorant et al. 2003; Peyrot et al. 2015; Cohen et al. 2020; Kendler et al. 2020; Rosoff et al. 2021). Participant-reported adulthood educational attainment at W4 was included as a categorical covariate with the following five levels: (1) did not complete high school (2), completed high school (3), completed some college/vocational/technical training (4), completed college/vocational/technical training, and (5) completed education beyond a bachelor’s degree.

#### Ancestry Principal Components

The Add Health study previously calculated genetic ancestry principal components using GRAF-pop (Jin et al. 2019). The first 10 principal components generated were included in analyses to adjust for confounding due to population stratification that may be produced from differences in allele frequencies across genetic ancestry groups. The first 10 principal components were chosen at the guidance of previously conducted PGS research in Add Health, which recommends using the first 10 principal components to adjust for population stratification based on ancestry-specific effects when analyzing multiple ancestry groups (Braudt and Harris 2020). These principal components were treated as continuous variables.

### Genotyping and Quality Control

Detailed information about the genotyping and quality control procedures of the genetic data from Add Health genetic data and the two discovery GWAS populations was provided elsewhere (Zhou et al. 2023; Meng et al. 2024). Importantly, all three data sources filtered individual samples for ambiguous biological sex, high levels of genotypic missingness, and high levels of heterozygosity. Each source also filtered variants with high genotypic missingness, low minor allele frequency, and breakage of Hardy–Weinberg equilibrium assumptions. The cut-off percentages and p value thresholds for these measures were not uniform across the three sources. The three studies also differed in the genotypic arrays and imputation protocols used, with some overlap.

High-quality SNPs common between Add Health and the discovery samples were included in the analyses to account for differences in quality control, genotypic measurement, and imputation methods. First, for ease of further analysis each of the output summary statistic files was standardized, ensuring that column names, data formats, and column order were identical across files, which was done using manual data manipulation in addition to the *MungeSumstats* R package (version 1.16.0) (Murphy et al. 2025). Second, through utilizing a reference SNP database [dbSNP build 144 (Sherry et al. 2001)] and reference genome [1000 Genomes GRCh37 (Auton et al. 2015)], multiple quality control filters and checks were also performed by the *MungeSumstats* package on each summary statistics file, including: removing duplicates, identifying and removing statistical outliers (p values < 5e^−324^), filtering by imputation score (INFO > 0.9, when available), filtering by minor allele frequency (MAF > 5%), removal of strand-ambiguous SNPs, removal of non-biallelic SNPs, ensuring consistent effect allele and reference alleles between files, and imputing missing information (such as sample size using the standard error of the beta and minor allele frequency or Z-score using the beta divided by the standard error of the beta). These additional quality control procedures described above were completed using the *MungeSumstats* package in R (Murphy et al. 2025).

### Genome-wide Polygenic Scores for MDD and AUD

Four PGS were calculated using PRS-CSx for each MDD and AUD (Ruan et al. 2022). The first PGS was meta-analyzed across the ancestry groups (META) and the other three PGS were generated specific to each ancestry group (AFR, AMR, EUR). These four PGS were generated by using the built-in meta-analysis function within PRS-CSx by including the meta-analysis flag (–meta) when calling the PRS-CSx function. PRS-CSx requires a linkage disequilibrium (LD) reference panel for each ancestry group, a discovery GWAS summary statistics file for each ancestry, a list of well-measured SNPs, and a list of target SNPs to calculate PGS weights specific to the target dataset. As such, this study used ancestry-specific reference panels from 1000 Genomes and a list of well-measured SNPs from HapMap Phase 3 to generate PGS for the target dataset based on the discovery datasets as described above (Altshuler et al. 2010; Auton et al. 2015). Each individual in Add Health was scored using the meta-analyzed PGS weights (META PGS) and their respective ancestry-specific PGS weights (EUR, AFR, and AMR PGS) using PLINK 1.9 (Chang et al. 2015). The distribution of META-MDD PGS and META-AUD PGS by case status can be observed in Supplemental Figs. 1 & 2. Resulting scores were standardized by ancestry group by subtracting the ancestry specific mean PGS value from the raw PGS values and then dividing by the ancestry specific standard deviation of PGS scores before being analyzed.

### Analytical Strategy

#### Univariate Tests of Covariates

Chi-square tests and one-way ANOVAs were used to test categorical and continuous covariates against the four outcome groups (Non-Affected, MDD-only, AUD-only, and Comorbid MDD-AUD), respectively.

#### Imputation of Covariates

The distribution of most covariates demonstrated a low degree of missingness (< 1.0%), with the exception of self-reported adulthood household income (5.8% missingness) and parent-reported adolescent household income (22.2% missingness). Consequently, missing values for adolescent household income and adulthood household income were imputed, while the remainder of missingness was removed from the dataset. Multiple imputations by chained equations (MICE) were used to generate 10 imputed datasets utilizing predicted mean matching over 50 iterations (van Buuren and Groothuis-Oudshoorn 2011). Predicted mean matching uses a subset of complete case data to create a regression predicting columns with missing values using all the other variables. This regression is used to predict the missing values. These predicted values are then matched to the complete datapoint with the closest predicted values, then the missing values are replaced with true values from that complete datapoint. This process is repeated for a specified number of iterations and imputations. Imputation was conducted using the *mice* package in R (version 3.17.0), under the assumption that adolescent and adult household income data were missing at random (van Buuren and Groothuis-Oudshoorn 2011). This assumption is appropriate as the missingness in adolescent and adult household income was found to be associated with the other covariates included in this analysis. Neither the PGS variables nor outcome variables for MDD, AUD, or comorbid MDD-AUD were used for the imputation.

#### Pearson Correlation between MDD and AUD PGS

Pearson correlations with 95% confidence intervals and p-values for the correlations, were calculated between the META-MDD PGS and META-AUD PGS within the whole population and within each of the ancestry specific populations using the cor.test() function from the *stats* package in R (version 4.5.2).

#### Tests of Association between PGS with MDD, AUD and Comorbid MDD-AUD

This analysis used logistic regression models adjusted for the covariates mentioned above. Models were estimated for each of the ten imputed datasets, and the results were pooled across the ten imputed datasets. For each outcome, four pooled logistic regression models were estimated: (M0) covariates only; (M1) META MDD PGS; (M2) META AUD PGS; and (M3) Joint use of META MDD and AUD PGS. In the cases where M3 were found to be the best fitting model, another model was evaluated to test whether the joint effects of the META MDD and META AUD PGS were qualitatively different from their additive effects by adding an interaction term between the two PGS. The assumption of linearity in the logit for continuous covariates (Age, Adolescent Household Income, and Adult Household Income) was evaluated through plotting predicted probabilities against the covariate values in the full META PGS models for each of the three outcomes. None of the continuous covariates violated the assumption of linearity in the logit. The focus of this analysis was to compare multiple models, following a traditional model building approach, using model fit metrics and assessing the amount of variance explained by PGS (Hosmer et al. 2013). As such inference is only drawn from the final models and no multiple testing corrections were conducted.

Comparisons of the amount of additional variance explained of each model containing a PGS of interest were calculated by subtracting the adjusted-R^2^ of the covariate-only model from the adjusted-R^2^ of the model that contained the PGS of interest. Wald p-values of the PGS estimates in each model and Likelihood Ratio Tests between the covariates-only and the other three models were used to determine the significance of PGS in the models at a significance level of *p* < 0.05. Model parsimony was evaluated using Akaike Information Criterion (AIC) and Bayesian Information Criterion (BIC). Pooled logistic regressions were estimated using the glm.mids() function from the *mice* package in R. All analyses were conducted in R (version 4.5.1).

#### Sensitivity Analyses

Three sensitivity analyses were conducted in this study. The first sensitivity analysis evaluated the degree to which the newly generated PGS improved the amount of variance explained by PGS compared to previously generated PGS in Add Health. This analysis was accomplished by repeating the analyses summarized above using prior EUR PGS that were previously generated in Add Health while applying the PRSice method for PGS generation (Euesden et al. 2014), using a broad depression GWAS (Howard et al. 2018) and alcohol dependence GWAS (Walters et al. 2018) for discovery samples (Braudt and Harris 2020). As the prior PGS conducted in Add Health for MDD and AUD were in EUR populations, the amount of variance explained, and the effect estimates of the prior PGS were then compared to those of the new EUR PGS instead of the META PGS.

The second sensitivity analysis evaluated the magnitude of possible population stratification bias that could occur when studying multiple ancestry groups with differences in outcome prevalence by ancestry group in a single model. The prevalence of each of the three outcomes significantly differed by ancestry group, MDD prevalence was found to be 11.4%, 8.7%, and 7.1% for EUR, AFR, and AMR samples, respectively; AUD prevalence was 31.4%, 14.7%, and 25.5% respectively; lastly comorbid MDD-AUD prevalence was found to be 8.9%, 2.3%, and 4.7% for EUR, AFR, and AMR samples, respectively. The sensitivity analysis was conducted as described above, but stratified for each of the three ancestry groups, using the ancestry-specific PGS instead of the META PGS. The amount of variance explained and effect estimates of the ancestry-specific PGS were compared to those of the META PGS in the initial analysis.

A third sensitivity analysis evaluated possible misclassification bias that would have been caused by not excluding participants based on their AUD, MDD, or comorbid MDD-AUD status from the outcome group definitions. Thus, the initial analysis was repeated with new outcome group definitions: (1) For the MDD outcome, individuals with AUD-only were included in the control group, and those with comorbid MDD-AUD were included as MDD cases; (2) For AUD, individuals with MDD-only were included in the control group, and those with comorbid MDD-AUD were included as AUD cases; (3) Finally, for the comorbid MDD-AUD outcome, those with MDD-only or AUD-only were included in the control group. Sample sizes of these new outcome groups are described in Supplemental Table 3. The amount of variance explained and effect estimates of the META PGS in this analysis were compared to those of the initial analysis.

## Results

### Population Characteristics

A total of 7,965 individuals from Add Health were included across the described analyses, including 4,454 healthy controls (55.9%), 822 MDD-only cases (10.3%), 2145 AUD-only cases (26.9%), and 544 comorbid MDD-AUD (6.8%) cases across all ancestry groups included. There was a total of 5218, 1836, and 911 EUR, AFR, and AMR individuals, respectively. The prevalence of each of the three outcomes significantly differed by ancestry group (Table 2).

**Table 2:** Outcome distribution by ancestry in add health

Case status	European	African	Admixed American
MDD (excluding AUD)	590 (11.3%)	162 (8.8%)	70 (7.7%)
Control	2522 (48.3%)	1368 (74.5%)	564 (61.9%)
Excluded	2106 (40.4%)	306 (16.7%)	277 (30.4%)
AUD (excluding MDD)	1645 (31.5%)	263 (14.3%)	237 (26.0%)
Control	2522 (48.3%)	1368 (74.5%)	564 (61.9%)
Excluded	1051 (20.2%)	205 (11.2%)	110 (12.1%)
Comorbid MDD-AUD (excluding single disorders)	461 (8.8%)	43 (2.3%)	40 (4.4%)
Control	2522 (48.3%)	1368 (74.5%)	564 (61.9%)
Excluded	2235 (42.9%)	425 (23.2%)	307 (33.7%)
Total	5218 (100%)	1836 (100%)	911 (100%)

Individuals were excluded from MDD analysis if they had any diagnosis of AUD. Individuals were excluded from AUD analysis if they had any diagnosis of MDD. Individuals were excluded from Comorbid MDD-AUD analyses if they only had one diagnosis of MDD or AUD

There were significant univariate associations between each covariate and all outcomes (MDD, AUD, and comorbid MDD-AUD) in the target (Add Health) sample (Table 3). Further, these associations remained significant in multivariate analyses including all covariates without any PGS (Table 4).

**Table 3:** Distribution of add health sample by outcome

Variable	Unaffected	MDD-only	AUD-only	Comorbid MDD-AUD
	*N* (%)	*N* (%)	*N* (%)	*N* (%)
Race/ethnicity*				
Asian	19 (0.4)	3 (0.4)	5 (0.2)	0 (0.0)
Black	1,345 (30.2)	156 (19.0)	259 (12.1)	43 (7.9)
Hispanic	554 (12.4)	69 (8.4)	220 (10.3)	34 (6.2)
Native American	9 (0.2)	6 (0.7)	2 (0.1)	4 (0.7)
White	2,527 (56.7)	588 (71.5)	1,659 (77.3)	463 (85.1)
Biological sex*				
Female	2,437 (54.7)	597 (72.6)	853 (39.8)	362 (66.5)
Male	2,017 (45.3)	225 (27.4)	1,310 (60.2)	182 (34.5)
Mean adolescent HHI (SD)*	4.25 (2.01)	4.29 (1.94)	4.83 (1.89)	4.83 (1.92)
Mean adulthood HHI (SD)*	5.11 (2.05)	4.49 (2.17)	5.57 (1.91)	4.98 (2.07)
Educational attainment*				
Did not complete high school	397 (8.9)	106 (12.9)	134 (6.2)	30 (5.5)
High school	828 (18.6)	171 (20.8)	320 (14.9)	56 (10.3)
Some college/vocational/technical training	1656 (37.2)	332 (40.4)	847 (39.5)	279 (51.3)
Completed college/vocational/technical training	1069 (24.0)	142 (17.3)	591 (27.6)	134 (24.6)
Graduate degree	504 (11.3)	71 (8.6)	253 (11.8)	45 (8.3)
Mean age at Wave 4 (SD)*	28.54 (1.79)	28.48 (1.80)	28.39 (1.76)	28.23 (1.77)
Total sample size	4454	822	2145	544

*MDD* major depressive disorder, *AUD* alcohol use disorder, *HHI* household income

Variables with an * are statistically different with a *p* < 0.05 for respective chi-square tests and one-way ANOVAs

**Table 4:** Covariates only (M0) models results

Variable name	MDD model OR (95% CI)	MDD model *p* value	AUD model OR (95% CI)	AUD model *p* value	Comorbid MDD-AUD model OR (95% CI)	Comorbid MDD-AUD model *p* value
R/E: White	Reference	–	Reference	–	Reference	**–**
R/E: Black	**0.41 (0.34–0.51)**	**1.49E-17**	**0.33 (0.28–0.38)**	**1.07E-45**	**0.16 (0.11–0.22)**	**1.11E-27**
R/E: Hispanic	**0.54 (0.41–0.72)**	**1.72E-05**	**0.64 (0.54–0.76)**	**6.17E-07**	**0.37 (0.25–0.54)**	**1.85E-07**
R/E: Asian	0.82 (0.18–3.72)	0.799	0.35 (0.12–1.06)	0.064	NA	NA
R/E: native American	**2.99 (1.01–8.87)**	**0.048**	0.29 (0.06–1.35)	0.115	1.99 (0.53–7.53)	0.3091
Sex: male	Reference	–	Reference	–	Reference	**–**
Sex: female	**2.29 (1.93–2.71)**	**1.37E-21**	**0.53 (0.47–0.59)**	**2.51E-29**	**1.59 (1.31–1.93)**	**3.12E-06**
Age at Wave 4	1.00 (0.96–1.05)	0.918	**0.95 (0.92–0.98)**	**0.001**	**0.93 (0.89–0.98)**	**0.009**
Adolescent HHI	1.04 (0.99–1.09)	0.128	**1.07 (1.04–1.10)**	**1.85E-05**	**1.09 (1.03–1.15)**	**0.003**
Adulthood HHI	**0.86 (0.82–0.90)**	**1.04E-10**	**1.04 (1.01–1.07)**	**0.022**	**0.89 (0.85–0.94)**	**8.46E-06**
EA: completed college/training	Reference	**–**	Reference	**–**	Reference	**–**
EA: did not complete HS	1.84 (1.35–2.51)	**1.32E-04**	**0.74 (0.58–0.95)**	**0.016**	0.69 (0.44–1.08)	0.102
EA: only completed HS	**1.51 (1.17–1.96)**	**0.002**	**0.77 (0.65–0.93)**	**0.005**	**0.60 (0.43–0.85)**	**0.004**
EA: attained some college/training	**1.47 (1.18–1.83)**	**0.001**	1.06 (0.92–1.22)	0.420	**1.47 (1.17–1.86)**	**0.001**
EA: graduate degree	1.00 (0.73–1.36)	0.989	0.96 (0.79–1.16)	0.661	**0.66 (0.46–0.95)**	**0.027**
A-PC 1	15.81 (0.09-2663.52)	0.291	2.72 (0.10-70.49)	0.547	0.14 (0.00-14.47)	0.405
A-PC 2	4.24 (0.02-819.04)	0.591	0.92 (0.02–40.11)	0.967	41.54 (0.02-91171.58)	0.342
A-PC 3	45.95 (0.90-2341.93)	0.056	2.36 (0.17–32.68)	0.521	7.31 (0.04-1303.66)	0.452
A-PC 4	0.62 (0.00-87.94)	0.850	1.33 (0.04–43.18)	0.873	185.04 (0.48-71117.12)	0.086
A-PC 5	0.19 (0.00-13.87)	0.451	2.47 (0.14–42.57)	0.534	0.12 (0.00-35.61)	0.468
A-PC 6	2.88 (0.04-184.89)	0.619	3.90 (0.23–67.14)	0.349	1.57 (0.00-596.73)	0.882
A-PC 7	28.55 (0.50-1630.65)	0.104	0.69 (0.04–12.54)	0.802	0.28 (0.00-58.08)	0.638
A-PC 8	0.66 (0.01–62.29)	0.859	0.71 (0.04–11.28)	0.809	1.05 (0.00-577.67)	0.989
A-PC 9	0.48 (0.01–21.98)	0.710	7.29 (0.21-259.27)	0.276	12.69 (0.01-14952.17)	0.481
A-PC 10	0.11 (0.00-17.57)	0.396	0.09 (0.00-1.81)	0.114	4.56 (0.00-9410.35)	0.697

Bolded estimates are statistically significant at *p* < 0.05

*MDD* major depressive disorder, *AUD* alcohol use disorder, *OR* odds ratio, *95% CI* 95% confidence interval, *R/E* race/ethnicity, *HHI* household income, *EA* educational attainment, *HS* high school, *A-PC* ancestry principal component

There was a moderate correlation between META-MDD PGS and META-AUD PGS in the whole population (*r* = 0.13; 95% CI 0.11–0.15; *p* < 2.20 × 10^− 16^). Within the EUR population, the correlation between META-MDD PGS and META-AUD PGS was *r* = 0.14 (95% CI 0.12–0.17; *p* < 2.20 × 10^− 16^), in the AFR population it was *r* = 0.08 (95% CI 0.04–0.13; *p* = 3.99 × 10^−4^), and in the AMR population it was *r* = 0.16 (95% CI 0.09–0.22; *p* = 1.47 × 10^−6^).

### PGS Outcome Prediction

#### MDD

META-MDD PGS was associated with MDD (Table 5; OR: 1.25; 95% CI 1.17–1.33; *p* = 2.05 × 10^−08^). This model, which included META-MDD PGS along with the covariates mentioned above, explained an additional 0.65% variance compared to the covariates-only model, as measured by McFadden Adjusted R^2^. In the model that included META-AUD PGS and covariates, META-AUD PGS was not associated with MDD (Table 5). This model explained no additional variance compared to a model that included covariates only. In the model that included MDD and AUD PGS in addition to covariates, META-MDD PGS was associated with MDD (Table 5; OR: 1.26; 95% CI 1.16–1.35; *p* = 2.69 × 10^− 06^), while META-AUD PGS was not associated with MDD. This model only explained an additional 0.63% of variance compared to just using the covariates, 0.02% less than the model that included META-MDD PGS and covariates.

**Table 5:** META PGS model estimates and performance

Model	McFadden adjusted *R*^2^ (%)	AIC	BIC	MDD PGS OR (95% CI)	MDD PGS *p*-value	AUD PGS OR (95% CI)	AUD PGS *p* value
*MDD*							
BASE-M0	5.22	4326.7	4477.8	–	–	–	–
META-PGS-M1	**5.87**	**4296.7**	**4454.4**	**1.25 (1.17–1.33)**	**2.05E-08**	–	–
META-PGS-M2	5.22	4326.4	4484.1	–	–	1.07 (0.98–1.15)	0.125
META-PGS-M3	5.85	4297.9	4462.2	1.24 (1.16–1.32)	4.55E-08	1.04 (0.95–1.12)	0.386
*AUD*							
BASE-M0	5.84	7833.9	7990.2	–	–	–	–
META-PGS-M1	5.82	7835.8	7998.9	1.01 (0.95–1.06)	0.789	–	–
META-PGS-M2	**7.36**	**7707.3**	**7870.3**	–	–	**1.37 (1.32–1.43)**	**1.25E-28**
META-PGS-M3	7.36	7707.8	7877.7	0.97 (0.91–1.02)	0.230	1.38 (1.32–1.44)	6.58E-29
*Comorbid MDD-AUD*							
BASE-M0	8.13	3155.4	3305.3	–	–	–	–
META-PGS-M1	9.27	3116.4	3272.8	1.35 (1.25–1.44)	2.54E-10	–	–
META-PGS-M2	12.41	3008.5	3164.9	–	–	1.82 (1.72–1.92)	1.03E-31
META-PGS-M3	**13.01**	**2988.1**	**3151.0**	**1.26 (1.16–1.35)**	**2.69E-06**	**1.77 (1.66–1.87)**	**3.49E-28**

The best performing model has been bolded for each outcome

*PGS* polygenic score, *META* meta-analyzed PRS-CSx PGS, *AIC* Akaike Information Criterion, *BIC* Bayesian Information Criterion, *OR* odds ratio, *MDD* major depressive disorder, *AUD* alcohol use disorder, M0 = Covariates Only, M1 = MDD PGS + Covariates, M2 = AUD PGS + Covariates, M3 = MDD PGS + AUD PGS + Covariates

#### AUD

META-MDD PGS was not significantly associated with AUD (Table 5). This model, which included META-MDD PGS along with covariates, explained 0.02% less variance of AUD compared to a model including only covariates. A model including META-AUD in addition to covariates detected an association with AUD (Table 5; OR: 1.37; 95% CI 1.32–1.43; *p* = 1.25 × 10^−28^). This model explained an additional 1.52% of variance compared to a model with just covariates. A model including META -AUD and -MDD PGS only detected an association with AUD for META-AUD PGS (Table 5; OR: 1.38; 95% CI 1.32–1.44; *p* = 6.58 × 10^−29^) but not for META-MDD PGS. This model explained an additional 1.52% of variance compared to a model including covariates, which is the same amount of variance explained by the model with just AUD PGS with covariates.

#### Comorbid MDD-AUD

The model that included META-MDD PGS along with covariates, when used alone, was associated with comorbid MDD-AUD (Table 5; OR: 1.35; 95% CI 1.25–1.43; *p* = 2.54 × 10^−10^). This model explained an additional 1.14% variance compared to the model that included covariates-only. The model that included META-AUD PGS along with covariates found that META-AUD PGS was associated with comorbid MDD-AUD (Table 5; OR: 1.82; 95% CI 1.72–1.92; *p* = 1.03 × 10^− 31^). This model explained an additional 4.28% variance compared to the model that included covariates-only. In the model that included both PGS along with covariates, META-MDD PGS (Table 5; OR: 1.26; 95% CI 1.16–1.35; *p* = 2.69 × 10^−6^) and META-AUD PGS (Table 5; OR: 1.77; 95% CI 1.66–1.87; *p* = 3.49 × 10^− 28^) were associated with comorbid MDD-AUD. The effect sizes were noticeably attenuated compared to when they were used singularly, as expected due to the genetic correlation between the two traits. This model explained an additional 4.88% variance compared to the model that included covariates-only, a larger improvement than the singular PGS models.

META-MDD and META-AUD PGS were uniquely associated with comorbid MDD-AUD when used separately and together. Therefore, we estimated a model that included an interaction term between the two PGS, to evaluate if their joint effect was qualitatively different than their additive effects. In this model, the effect estimates for the META-MDD PGS (OR: 1.26; 95% CI 1.16–1.36; *p* = 4.18 × 10^−6^) and META-AUD PGS (OR: 1.77; 95% CI 1.66–1.88; *p* = 1.45 × 10^−27^) were slightly changed, and the interaction term was not statistically significant (OR: 0.99; 95% CI 0.89–1.09; *p* = 0.81).

### Sensitivity Analysis

#### Comparison to Prior PGS in Add Health

To evaluate the quality of the PGS generated for this study, MDD and Alcohol Dependence PGS that were included in the Add Health data were used in a repeated analysis instead of the META PGS (Braudt and Harris 2020). As these prior PGS were created using EUR-only GWAS summary statistics, they were compared to EUR-MDD PGS and EUR-AUD PGS from the ancestry-specific sensitivity analysis. When comparing the EUR-MDD PGS to the previous MDD PGS, a slight improvement was observed in the EUR-MDD PGS over the previous MDD PGS, explaining 0.16% more variance of MDD and 0.52% more variance of comorbid MDD-AUD (Supplemental Table 4). The EUR-AUD PGS largely improved over the previous alcohol dependence PGS. The prior alcohol dependence PGS was not found to be significantly associated with the outcome of any model estimated, while the EUR-AUD PGS was significantly associated with MDD, AUD, and comorbid MDD-AUD. The EUR-AUD PGS explained 0.29%, 3.43%, and 6.61% more variance than the previous alcohol dependence PGS for MDD, AUD, and comorbid MDD-AUD, respectively.

#### Ancestry Specific PGS Prediction

Results for the EUR PGS differed slightly compared to the META PGS results (Supplemental Table 4). Specifically, the EUR-MDD PGS was associated with MDD, and the EUR-AUD PGS was significantly associated with AUD, and both were associated with comorbid MDD-AUD. The EUR-AUD PGS was also significantly associated with MDD (OR: 1.18; 95% CI 1.07–1.29; *p* = 0.002) and using MDD and AUD PGS together performed better than either PGS alone for MDD, explaining an additional 0.1% variance than the with the MDD PGS and covariates. Using both PGS was still the best for predicting comorbid MDD-AUD. When looking at the AFR- and AMR-specific PGS results, neither of the ancestry-specific analyses were powered enough to detect significant PGS results (Supplemental Tables 5–6).

#### Non-excluded Case Group Analysis

An additional sensitivity analysis was conducted where individuals with a diagnosis of the other respective disorder were not excluded from the MDD or AUD analyses, and where individuals with only one diagnosis were not excluded from the control group for predicting comorbid MDD-AUD (Supplemental Table 3). Using these case definitions, the amount of variance explained in the covariate-only models of MDD, AUD, and comorbid MDD-AUD were 5.82%, 5.64%, and 5.19% respectively (Supplemental Table 7). For MDD, the best performing model was the model that included both META-MDD PGS (OR: 1.27; 95% CI 1.21–1.33; *p* = 2.60 × 10^− 14^) and META-AUD PGS (OR: 1.13; 95% CI 1.06–1.19; *p* = 1.54 × 10^−4^). For AUD, the best-performing model included just the META-AUD PGS (OR: 1.42; 95% CI 1.37–1.47; *p* = 6.88 × 10^−42^) alongside covariates. For MDD and AUD, using these case definitions, more variance was explained by PGS and the magnitudes of PGS associations were inflated compared to the initial MDD-only or AUD-only case definition models. For the comorbid MDD-AUD models using these non-exclusion outcome definitions, the best performing model was still the one that utilized both META MDD-PGS (OR: 1.25; 95% CI 1.16–1.34; *p* = 1.46 × 10^−6^) and META AUD-PGS (OR: 1.50; 95% CI 1.40–1.59; *p* = 2.28 × 10^−18^). In the comorbid MDD-AUD models, the amount of variance explained was lower and the magnitudes of PGS associations were reduced compared to the initial analysis.

## Discussion

To our knowledge, this is the first study to investigate the use of PGS for MDD and AUD to predict MDD, AUD, and comorbid MDD-AUD. Results from this study indicate that: (1) the use of both META-MDD and -AUD PGS improved prediction of comorbid MDD-AUD compared to a PGS from a single trait; (2) use of both PGS did not improve the prediction of either MDD or AUD; (3) more variance was explained in comorbid MDD-AUD when using any set of PGS compared to either MDD or AUD; and (American ) the effect sizes of PGS were much larger in comorbid MDD-AUD models compared to either MDD and AUD models.

### Joint Use of MDD and AUD PGS Produced Mixed Prediction Improvement

The use of genetically correlated PGS did not consistently improve the amount of variance explained by genetic effects in the initial analysis. For example, the PGS for META-MDD and META-AUD were uniquely associated with comorbid MDD-AUD when used separately or together. In contrast, for MDD or AUD, only the same-trait META PGS were uniquely associated with the outcomes and no cross-trait associations (e.g., META PGS for MDD with AUD) were significant. Additionally, the results were not consistent in either the EUR ancestry stratified sensitivity analysis or the non-exclusionary outcome definition sensitivity analysis where the EUR-AUD and META-AUD PGS were associated with MDD when used singularly or jointly with the MDD PGS.

To date, no studies have evaluated the predictive ability of genetically correlated PGS in comorbid MDD-AUD. However, other studies of psychiatric and substance use disorder comorbidities report that models improved by including shared genetic effects (Strom et al. 2021; Shi et al. 2023; Ribasés et al. 2023; Tabrizi et al. 2025). It is possible that no significant cross-trait PGS associations were detected in the initial analysis for reasons related to study design, including: (1) bias induced by population stratification and ancestral group differences in PGS quality; (2) exclusion of comorbid individuals from the single disorder models who are usually the most severe MDD/AUD cases, which reduced the magnitude of associations between the PGS and the outcomes; (3) weak predictive ability of META-MDD PGS compared to the META-AUD PGS, leading to no cross-trait effects for MDD PGS.

The results of the single trait analyses in this study are also somewhat different from other studies. Specifically, for MDD and AUD, studies have demonstrated that use of MDD PGS alone improved the prediction of alcohol phenotypes, which was not replicated in this study (Andersen et al. 2017; Karpyak et al. 2023). In some of these studies, comorbid patients within the study samples were not excluded in the single trait analyses (Jansen et al. 2021; Albiñana et al. 2022, Karpyak et al. 2023). Similar to the current study, others have concluded that when comorbid individuals are excluded from the prediction of a single disorder, the predictive ability of cross-trait PGS effects is reduced (Andersen et al. 2017). These results support the concept that individuals who have higher odds of having a comorbid MDD-AUD diagnosis are associated with specific biological subtypes of MDD and AUD (Østergaard et al. 2011; Tang et al. 2025; Müller et al. 2020; Zhu et al. 2022; Brière et al. 2014; McHugh and Weiss 2019).

### More Variance Explained and Larger Effect Estimates in Comorbid MDD-AUD than Either MDD or AUD

Across all analyses, the amount of variance explained by PGS in the comorbid MDD-AUD models was greater than single-trait models of MDD or AUD. Further, the effect sizes of the PGS odds ratios were larger in the best performing comorbid MDD-AUD model compared to either of the best performing MDD or AUD models. Additionally, the magnitude of the interaction between MDD and AUD common genetic effects was not statistically significant. These results together suggest that individuals with comorbid MDD-AUD are more likely to have a higher genetic load for MDD and AUD, but do not have a different genetic architecture than those with a single trait. These results also suggest that common genetic effects play a larger role in comorbid MDD-AUD than in either MDD or AUD. Consequently, the results of this study provide evidence towards comorbid MDD-AUD being a more genetically severe subtype of MDD and AUD. Other studies that have evaluated the use of genetically-correlated PGS for psychiatric comorbidities demonstrate that higher single trait PGS score values are associated with higher levels of comorbidity and comorbid groups tend to have larger amounts of variances explained by common genetic effects than the single traits (Strom et al. 2021; Shi et al. 2023; Ribasés et al. 2023; Tabrizi et al. 2025).

### Newly Generated PGS Improve the Amount of Variance Explained Compared to Prior PGS in Add Health

In the first sensitivity analysis, the newly generated EUR PGS explain more variance than the prior PGS of MDD and alcohol dependence. While there are no previous estimates of PGS reported to compare against for the META PGS in Add Health, it can be assumed that the META PGS also improved alongside the EUR PGS. This was expected as the new PGS used larger sample sizes and were generated using stronger and more sensitive methods (Ruan et al. 2022; Zhou et al. 2023; Meng et al. 2024). The degree of improvement was not consistent for EUR-MDD PGS and EUR-AUD PGS, the AUD PGS was greatly improved and the MDD was only slightly improved. This sensitivity analysis demonstrates the importance of continuing to collect larger and more representative genetic samples and to continue utilizing methods that are more sensitive to ancestral differences through the improvement in these PGS.

### Residual Population Stratification Bias May Influence the Effects of Genetically Correlated PGS

The second sensitivity analysis was stratified by ancestry group and used ancestry-specific PGS to evaluate the degree to which the results of the initial cross-ancestry analysis using META PGS could be subject to bias from unadjusted population stratification. In the EUR analysis, the results were smaller compared to the initial analysis. While the AFR and AMR analyses were heavily impacted by the reduction in sample size and did not have sufficient statistical power to detect any associations. Additionally, the correlations between META-MDD and META-AUD PGS differed by ancestry, suggesting that common genetic effects implicated in MDD, AUD, and their comorbidity may differ by ancestry. This may also indicate that uncontrolled confounders associated with population stratification could be inflating the amount of variance explained, increasing the probability of spurious associations in the initial analysis (Chan et al. 2023; Grinde et al. 2024). Other studies investigating the utilization of shared genetic effects in single or comorbid psychiatric and substances used disorders have often been limited to studying this effect in European ancestry groups only, to eliminate this possible confounding through population stratification (Andersen et al. 2017; Jansen et al. 2021; Albiñana et al. 2022; Karpyak et al. 2023; Strom et al. 2021; Shi et al. 2023; Tabrizi et al. 2025). These results therefore offer preliminary evidence encouraging additional work that includes data on shared genetic effects across comorbid and single-trait outcomes to include data from multiple ancestral populations.

### The Predictive Ability of PGS May be Affected by Outcome Misclassification due to Comorbidity

The degree to which results would have been affected by misclassification bias was evaluated by comparing the results of the initial analysis to an analysis that did not exclude participants based on case status. The inclusion of participants with MDD in the AUD models and vice versa increased the magnitudes of PGS associations and tightened the ranges of their respective 95% CIs, suggesting inflated estimates of association. The presence of tighter confidence intervals and increased variance explained was unexpected since the misclassification was expected to increase the heterogeneity in the outcome measures. Additionally, the misclassification in the single trait models identified a possibly inflated cross-trait association of META-AUD PGS with MDD that differed from the initial analysis. The inflation seen in these models may have been caused by the inclusion of the participants who also had comorbid MDD-AUD and as such had greater severity. Therefore, it is possible that the model is not necessarily performing better. Instead, it is measuring a different conceptual outcome where comorbid patients (who have a larger association between PGS and the outcome) are not treated differently than those with a single trait. Ultimately, these results emphasize the need for clarity in definitions of cases vs. controls, particularly for single-trait analyses.

For the comorbid MDD-AUD models, the sensitivity analysis explained much less variance, had smaller magnitudes of association, and had wider 95% CIs than the initial analysis. This is likely due the fact that those with a genetically correlated outcome (MDD or AUD) were included in the control group. In the comorbid MDD-AUD analysis, there are no observed benefits to including those with single traits in the control group, as they are more similar to those with comorbid MDD-AUD, resulting in a large amount of misclassification bias. Overall, the exclusion of participants based on their single or comorbid disorder case status produces more conservative and less biased results.

### Study Limitations

The reported results and interpretations should be considered in light of the following limitations. First, the effect size of the PGS could have been biased due to differences in outcome prevalence and allele frequency between the discovery GWAS and Add Health samples. Nevertheless, extensive QC methods for each data source and selection of the PRS-CSx method to control for differences in MAF of SNPs across the genome and across ancestry groups were conducted. Further, analyses were also controlled for population stratification by adjusting for ancestry PCs, and other related covariates in all models.

Second, estimates of PGS effect sizes could have been affected by differences in MDD and AUD outcome measurement between Add Health and the discovery GWAS. MDD was measured as a self-reported diagnosis of depression in Add Health, while the discovery GWAS combined multiple diagnosis methods of MDD. Nevertheless, there is approximately 60–90% concordance between structured interviews and self-report measures in MDD (Sanchez-Villegas et al. 2008; Stuart et al. 2014; Davies et al. 2022). AUD in Add Health was measured using a DSM-IV checklist, while the discovery GWAS combined multiple diagnosis methods into one AUD variable. The definition of AUD in Add Health included DSM IV definitions of both alcohol dependence and alcohol abuse, which was selected as it is more similar to the DSM-5 definition of AUD. However, the inclusion of participant affected with alcohol abuse could cause an overestimation of AUD prevalence when compared to the discovery AUD GWAS which primarily included individuals with DSM-IV alcohol dependence and a smaller proportion of individuals with DSM-IV alcohol abuse, which possibly induce bias to the AUD PGS estimates in this analysis. However, the combination of these AUD phenotypes is supported by high genetic correlations (r_g_) found between these traits (AUD – AD r_g_: 0.98) (Zhou et al. 2020).

Third, the definition of AUD in this study included participants in Add Health who have never drunk alcohol as controls, in addition to those who tried alcohol once and abstained afterward. Inclusion of these participants may reduce power and the effect size of AUD PGS for predicting AUD and in part comorbid MDD-AUD, as they could not develop AUD despite their genetic liability (Gupta et al. 2020). However, for this analysis, these participants were retained as the focus of this study was not only on AUD but also on MDD and comorbid MDD-AUD and removing these participants would have significantly reduce the study power.

Fourth, lifetime comorbid MDD-AUD represents participants whose MDD and AUD occurred within the same timeframe in addition to participants whose MDD and AUD could have occurred at different times. This introduced heterogeneity into the outcome measures and possibly affected the magnitude of PGS associations, given the more severe symptomatic and genetic nature of comorbid MDD-AUD patients.

Fifth, the age of the participants at the time of the outcome measurement in the target sample (Add Health) could have multiple downstream effects on the results of this study. The magnitude of PGS odds ratio estimates in lifetime MDD, AUD, and comorbid MDD-AUD could have been lower due to the younger age of the target sample cohort at the time of collection (24–32 years old at Wave 4, average = 28 years) compared to the discovery sample where the average age of participants differed by the cohorts included (14–63 years old on average). An earlier age of assessment of MDD and AUD could produce bias caused by misclassification of participants who would later develop the single or comorbid disorders. This issue also extends to estimates for educational attainment. Participants may not have achieved their highest level of educational attainment at the time of data collection. Nevertheless, the risk for AUD and MDD is highest in young adulthood, with individuals 18–25 years old having the highest prevalence for both AUD and MDD (Substance Abuse and Mental Health Services Administration 2022). Most AUD and MDD cases in Add Health are expected to be detected by the time they were assessed at W4, when the participants were between the ages of 24–32 (Seeley et al. 2019; Villarroel and Terlizzi 2020). Additionally, the analyses included age as a covariate, which should account for some of the possible bias caused by the limitations discussed above.

Sixth, there were an unknown number of overlapping participants between the sample populations of the two discovery GWAS used to generate PGS in this analysis (i.e., UK Biobank, Million Veterans Program, etc.). While it is not yet clear what sort of biases could be observed when using the PGS of two traits together that were generated from discovery GWAS with partially overlapping samples that could be extended to the results produced in Add Health.

## Conclusion

The use of PGS for MDD and AUD together strengthened the prediction of comorbid MDD-AUD, and in the prediction of MDD in some cases. This study demonstrates the benefits of continued research into leveraging shared genetic effects between MDD and AUD to strengthen the use of PGS in MDD, AUD, and comorbid MDD-AUD. Additionally, this study also demonstrated the greater role of common genetic effects in individuals with comorbid MDD-AUD compared to those with only MDD or AUD, indicating that the comorbidity between complex traits such as MDD and AUD may possibly be more genetically severe from the single disorders. The PGS generated in this study explain a small proportion of variance for these conditions, limiting their use for diagnosis of AUD, MDD, or comorbid AUD-MDD in clinical settings. However, this study provides an example of how genetically correlated PGS could be used to study a single disorder as well as its comorbidity with other conditions. This type of detail could help clinicians and patients in the future understand the role of genetic factors in comorbidity and potentially improve the clinical decision-making process. Future work is needed to investigate the role of genetic effects of MDD, AUD, and comorbid MDD-AUD in the context of the environmental risk factors to understand more about the distinctions between these three conditions.

## Supplementary Information

Below is the link to the electronic supplementary material.

Supplementary Material 1

## Data Availability

There are three sources for the data used in the preparation of this article. The Restricted-Use National Longitudinal Study of Adolescent to Adult Health (AddHealth) dataset was obtained through dbGaP (Study Accession: phs001367.v1.p1). Instructions on gaining access to Add Health restricted use data can be found at: [https://data.cpc.unc.edu/projects/2/view](https:/data.cpc.unc.edu/projects/2/view). The Million Veteran Program alcohol use disorder summary statistics for the Admixed American ancestry group were obtained through dbGaP (Study Accession: phs001672.v12.p1). Finally, the remaining ancestry-specific summary statistics for alcohol use disorder and major depressive disorder are openly available from the Psychiatric Genetics Consortium website: [https://pgc.unc.edu/for-researchers/download-results/](https:/pgc.unc.edu/for-researchers/download-results).

## References

[CR1] Albiñana C, Zhu Z, Schork AJ, Ingason A, Aschard H, Brikell I, Bulik CM, Petersen LV, Agerbo E, Grove J, Nordentoft M, Hougaard DM, Werge T, Børglum AD, Mortensen PB, McGrath JJ, Neale BM, Privé F, Vilhjálmsson BJ (2022) Multi-PGS enhances polygenic prediction: weighting 937 polygenic scores. medRxiv. 10.1101/2022.09.14.22279940

[CR2] Als TD, Kurki M, Grove J, Voloudakis G, Therrien K et al (2022) Identification of 64 new risk loci for major depression, refinement of the genetic architecture and risk prediction of recurrence and comorbidities. medRxiv. 10.1101/2022.08.24.22279149

[CR3] Altshuler DM, Gibbs RA, Peltonen L, Schaffner SF, Yu F et al (2010) Integrating common and rare genetic variation in diverse human populations. Nat 2010 467:7311. 10.1038/nature09298 10.1038/nature09298 PMC3173859 20811451

[CR4] American Psychiatric Association (2013) Diagnostic and statistical manual of mental disorders. American Psychiatric Association, Washington. 10.1176/APPI.BOOKS.9780890425596

[CR5] Andersen AM, Pietrzak RH, Kranzler HR, Ma L, Zhou H et al (2017) Polygenic Scores for Major Depressive Disorder and Risk of Alcohol Dependence. JAMA Psychiatry 74:1153–1160. 10.1001/JAMAPSYCHIATRY.2017.2269 28813562 10.1001/jamapsychiatry.2017.2269 PMC5710224

[CR6] Auton A, Abecasis GR, Altshuler DM, Durbin RM, Bentley DR et al (2015) A global reference for human genetic variation. Nat 2015 526:7571. 10.1038/nature15393 10.1038/nature15393 PMC4750478 26432245

[CR7] Bailey AJ, Quinn PD, McHugh RK (2025) Implications of the shift to DSM-5 for alcohol use disorder prevalence estimates in the National Survey on Drug Use and Health. Addiction 120:184–188. 10.1111/ADD.16670 39252673 10.1111/add.16670

[CR8] Barr PB, Ksinan A, Su J, Johnson EC, Meyers JL, Wetherill L, Latvala A, Aliev F, Chan G, Kuperman S, Nurnberger J, Kamarajan C, Anokhin A, Agrawal A, Rose RJ, Edenberg HJ, Schuckit M, Kaprio J, Dick DM (2020) Using polygenic scores for identifying individuals at increased risk of substance use disorders in clinical and population samples. Transl Psychiatry. 10.1038/S41398-020-00865-8 32555147 10.1038/s41398-020-00865-8 PMC7303212

[CR9] Benny C, Patte KA, Veugelers PJ, Senthilselvan A, Leatherdale ST, Pabayo R (2023) A Longitudinal Study of Income Inequality and Mental Health Among Canadian Secondary School Students: Results From the Cannabis, Obesity, Mental Health, Physical Activity, Alcohol, Smoking, and Sedentary Behavior Study (2016–2019). J Adolesc Health 73:70–78. 10.1016/J.JADOHEALTH.2023.01.028 37031091 10.1016/j.jadohealth.2023.01.028

[CR10] Blanco C, Alegría AA, Liu SM, Secades-Villa R, Sugaya L, Davies C, Nunes EV (2012) Differences among major depressive disorder with and without co-occurring substance use disorders and substance-induced depressive disorder: results from the National Epidemiologic Survey on Alcohol and Related Conditions. J Clin Psychiatry 73:865–873. 10.4088/JCP.10M06673 22480900 10.4088/JCP.10m06673

[CR11] Bourque VR, Poulain C, Proulx C, Moreau CA, Joober R, Forgeot d’Arc B, Huguet G, Jacquemont S (2024) Genetic and phenotypic similarity across major psychiatric disorders: a systematic review and quantitative assessment. Translational Psychiatry 2024 14(1 14):1–10. 10.1038/s41398-024-02866-3 10.1038/s41398-024-02866-3 PMC10981737 38555309

[CR12] Braudt DB, Harris KM (2020) Polygenic Scores (PGSs) in the National Longitudinal Study of Adolescent to Adult Health (Add Health) – Release 2. Carolina Population Center, University of North Carolina at Chapel Hill, Chapel Hill. 10.17615/9g92-vc17

[CR13] Brière FN, Rohde P, Seeley JR, Klein D, Lewinsohn PM (2014) Comorbidity between major depression and alcohol use disorder from adolescence to adulthood. Compr Psychiatry 55:526. 10.1016/J.COMPPSYCH.2013.10.007 24246605 10.1016/j.comppsych.2013.10.007 PMC4131538

[CR15] Cerdá M, Johnson-Lawrence VD, Galea S (2011) Lifetime income patterns and alcohol consumption: Investigating the association between long- and short-term income trajectories and drinking. Soc Sci Med 73:1178. 10.1016/J.SOCSCIMED.2011.07.025 21890256 10.1016/j.socscimed.2011.07.025 PMC3185179

[CR17] Chan TF, Rui X, Conti DV, Fornage M, Graff M, Haessler J, Haiman C, Highland HM, Jung SY, Kenny E, Kooperberg C, Marchland L, Le, North KE, Tao R, Wojcik G, Gignoux CR, Consortium P, Chiang CWK, Mancuso N (2023) Estimating heritability explained by local ancestry and evaluating stratification bias in admixture mapping from summary statistics. bioRxiv. 10.1101/2023.04.10.536252 37875120 10.1016/j.ajhg.2023.09.012 PMC10645552

[CR16] Chang CC, Chow CC, Tellier LCAM, Vattikuti S, Purcell SM, Lee JJ (2015) Second-generation PLINK: rising to the challenge of larger and richer datasets. Gigascience 4:7. 10.1186/S13742-015-0047-8 25722852 10.1186/s13742-015-0047-8 PMC4342193

[CR18] Chebib J, Guillaume F (2021) Pleiotropy or linkage? Their relative contributions to the genetic correlation of quantitative traits and detection by multitrait GWA studies. Genetics 219:iyab159. 10.1093/GENETICS/IYAB159 34849850 10.1093/genetics/iyab159 PMC8664587

[CR19] Chesmore K, Bartlett J, Williams SM (2018) The ubiquity of pleiotropy in human disease. Hum Genet 137:39–44. 10.1007/S00439-017-1854-Z 29164333 10.1007/s00439-017-1854-z

[CR20] Cohen AK, Nussbaum J, Weintraub MLR, Nichols CR, Yen IH (2020) Association of adult depression with educational attainment, aspirations, and expectations. Prev Chronic Dis. 10.5888/PCD17.200098 32857033 10.5888/pcd17.200098 PMC7478148

[CR21] Collins SE (2016) Associations between socioeconomic factors and alcohol outcomes. Alcohol Res 38:83 27159815 10.35946/arcr.v38.1.11 PMC4872618

[CR22] Conner KR, Gamble SA, Bagge CL, He H, Swogger MT, Watts A, Houston RJ (2014) Substance-induced depression and independent depression in proximal risk for suicidal behavior. J Stud Alcohol Drugs 75:567–572. 10.15288/JSAD.2014.75.567 24988255 10.15288/jsad.2014.75.567 PMC9798469

[CR23] Davies MR, Buckman JEJ, Adey BN, Armour C, Bradley JR et al (2022) Comparison of symptom-based versus self-reported diagnostic measures of anxiety and depression disorders in the GLAD and COPING cohorts. J Anxiety Disord 85:102491. 10.1016/J.JANXDIS.2021.102491 34775166 10.1016/j.janxdis.2021.102491

[CR25] Davis L, Uezato A, Newell JM, Frazier E (2008) Major depression and comorbid substance use disorders. Curr Opin Psychiatry 21:14–18. 10.1097/YCO.0B013E3282F32408 18281835 10.1097/YCO.0b013e3282f32408

[CR24] Davis LL, Wisniewski SR, Howland RH, Trivedi MH, Husain MM, Fava M, McGrath PJ, Balasubramani GK, Warden D, Rush AJ (2010) Does comorbid substance use disorder impair recovery from major depression with SSRI treatment? An analysis of the STAR*D level one treatment outcomes. Drug Alcohol Depend 107:161–170. 10.1016/J.DRUGALCDEP.2009.10.003 19945804 10.1016/j.drugalcdep.2009.10.003

[CR26] Dawson DA, Goldstein RB, Grant BF (2013) Differences in the profiles of DSM-IV and DSM-5 alcohol use disorders: implications for clinicians. Alcohol Clin Exp Res. 10.1111/J.1530-0277.2012.01930.X 22974144 10.1111/j.1530-0277.2012.01930.x PMC3800556

[CR27] Ellingson JM, Richmond-Rakerd LS, Statham DJ, Martin NG, Slutske WS (2016) Most of the genetic covariation between major depressive and alcohol use disorders is explained by trait measures of negative emotionality and behavioral control. Psychol Med 46:2919. 10.1017/S0033291716001525 27460396 10.1017/S0033291716001525 PMC9361478

[CR28] Ellis CA, Oliver KL, Harris RV, Ottman R, Scheffer IE, Mefford HC, Epstein MP, Berkovic SF, Bahlo M (2024) Inflation of polygenic risk scores caused by sample overlap and relatedness: Examples of a major risk of bias. Am J Hum Genet 111:1805–1809. 10.1016/J.AJHG.2024.07.014 39168121 10.1016/j.ajhg.2024.07.014 PMC11393675

[CR29] Euesden J, Lewis CM, O’Reilly PF (2014) PRSice: Polygenic Risk Score software. Bioinformatics 31:1466. 10.1093/BIOINFORMATICS/BTU848 25550326 10.1093/bioinformatics/btu848 PMC4410663

[CR30] Feinstein AR (1970) THE PRE-THERAPEUTIC CLASSIFICATION OF CO-MORBIDITY IN CHRONIC DISEASE. J Chronic Dis 23:455–468. 10.1016/0021-9681(70)90054-8 26309916 10.1016/0021-9681(70)90054-8

[CR31] First MB (2005) Mutually exclusive versus co-occurring diagnostic categories: the challenge of diagnostic comorbidity. Psychopathology 38:206–210. 10.1159/000086093 16145276 10.1159/000086093

[CR32] Grant BF, Goldstein RB, Saha TD, Patricia Chou S, Jung J, Zhang H, Pickering RP, June Ruan W, Smith SM, Huang B, Hasin DS (2015) Epidemiology of DSM-5 Alcohol Use Disorder: Results From the National Epidemiologic Survey on Alcohol and Related Conditions III. JAMA Psychiatry 72:757. 10.1001/JAMAPSYCHIATRY.2015.0584 26039070 10.1001/jamapsychiatry.2015.0584 PMC5240584

[CR33] Greenberg PE, Fournier AA, Sisitsky T, Simes M, Berman R, Koenigsberg SH, Kessler RC (2021) The economic burden of adults with major depressive disorder in the United States (2010 and 2018). PharmacoEconomics 39:653. 10.1007/S40273-021-01019-4 33950419 10.1007/s40273-021-01019-4 PMC8097130

[CR34] Grinde KE, Browning BL, Reiner AP, Thornton TA, Browning SR (2024) Adjusting for principal components can induce spurious associations in genome-wide association studies in admixed populations. bioRxiv. 10.1101/2024.04.02.587682 39680601 10.1371/journal.pgen.1011242 PMC11684764

[CR35] Gupta I, Dandavate R, Gupta P, Agrawal V, Kapoor M (2020) Recent advances in genetic studies of alcohol use disorders. Curr Genet Med Rep 8:27. 10.1007/S40142-020-00185-9 33344068 10.1007/s40142-020-00185-9 PMC7748121

[CR36] Harris KM, Halpern CT, Whitsel EA, Hussey JM, Killeya-Jones LA, Tabor J, Dean SC (2019) Cohort profile: The National Longitudinal Study of Adolescent to Adult Health (Add Health). Int J Epidemiol 48:1415–1415k. 10.1093/IJE/DYZ115 31257425 10.1093/ije/dyz115 PMC6857761

[CR37] Hasin D, Liu X, Nunes E, McCloud S, Samet S, Endicott J (2002) Effects of major depression on remission and relapse of substance dependence. Arch Gen Psychiatry 59:375–380. 10.1001/ARCHPSYC.59.4.375 11926938 10.1001/archpsyc.59.4.375

[CR38] Hasin DS, Sarvet AL, Meyers JL, Saha TD, Ruan WJ, Stohl M, Grant BF (2018) Epidemiology of adult DSM-5 major depressive disorder and its specifiers in the United States. JAMA Psychiatry 75:336–346. 10.1001/JAMAPSYCHIATRY.2017.4602 29450462 10.1001/jamapsychiatry.2017.4602 PMC5875313

[CR40] Hosmer D, Lemeshow S, Sturdivant RX (2013) Applied logistic regression, 3rd ed, Wiley, Chichester. 10.1002/9781118548387

[CR39] Howard DM, Adams MJ, Shirali M, Clarke TK, Marioni RE, Davies G, Coleman JRI, Alloza C, Shen X, Barbu MC, Wigmore EM, Gibson J, Hagenaars SP, Lewis CM, Ward J, Smith DJ, Sullivan PF, Haley CS, Breen G, Deary IJ, McIntosh AM (2018) Genome-wide association study of depression phenotypes in UK Biobank identifies variants in excitatory synaptic pathways. Nat Commun 2018 9(1 9):1–10. 10.1038/s41467-018-03819-3 10.1038/s41467-018-05310-5 PMC6117285 30166530

[CR41] Jansen AG, Jansen PR, Savage JE, Kraft J, Skarabis N, Polderman TJC, Dieleman GC (2021) The predictive capacity of psychiatric and psychological polygenic risk scores for distinguishing cases in a child and adolescent psychiatric sample from controls. J Child Psychol Psychiatry 62:1079–1089. 10.1111/JCPP.13370 33825194 10.1111/jcpp.13370 PMC8453516

[CR42] Jee YH, He Y, Lu W, Shi Y, Lazarev D, Daly MJ, Reeve MP, Martin AR (2025) Dissecting pleiotropy to gain mechanistic insights into human disease. Nat Rev Genet 2025:1–14. 10.1038/s41576-025-00908-0 10.1038/s41576-025-00908-0 41315867

[CR43] Jin Y, Schaffer AA, Feolo M, Holmes JB, Kattman BL (2019) GRAF-pop: a fast distance-based method to infer subject ancestry from multiple genotype datasets without principal components analysis. G3 (Bethesda). 10.1534/G3.118.200925 31151998 10.1534/g3.118.200925 PMC6686921

[CR44] Karpyak VM, Coombes BJ, Geske JR, Pazdernik VM, Schneekloth T, Kolla BP, Oesterle T, Loukianova LL, Skime MK, Ho AMC, Ngo Q, Skillon C, Ho MF, Weinshilboum R, Biernacka JM (2023) Genetic predisposition to major depressive disorder differentially impacts alcohol consumption and high-risk drinking situations in men and women with alcohol use disorder. Drug Alcohol Depend 243:109753. 10.1016/J.DRUGALCDEP.2022.109753 36608483 10.1016/j.drugalcdep.2022.109753 PMC9869363

[CR47] Kendler KS, Heath AC, Neale MC, Kessler RC, Eaves LJ (1993) Alcoholism and major depression in women. A twin study of the causes of comorbidity. Arch Gen Psychiatry 50:690–698. 10.1001/ARCHPSYC.1993.01820210024003 8357294 10.1001/archpsyc.1993.01820210024003

[CR46] Kendler KS, Davis CG, Kessler RC (1997) The familial aggregation of common psychiatric and substance use disorders in the National Comorbidity Survey: a family history study. Br J Psychiatry 170:541–548. 10.1192/BJP.170.6.541 9330021 10.1192/bjp.170.6.541

[CR48] Kendler KS, Ohlsson H, Fagan AA, Lichtenstein P, Sundquist J, Sundquist K (2020) Nature of the causal relationship between academic achievement and the risk for alcohol use disorder. J Stud Alcohol Drugs 81:446. 10.15288/JSAD.2020.81.446 32800080 10.15288/jsad.2020.81.446 PMC7437558

[CR49] Kessler RC, Avenevoli S, McLaughlin KA, Green JG, Lakoma MD, Petukhova M, Pine DS, Sampson NA, Zaslavsky AM, Merikangas KR (2012) Lifetime co-morbidity of DSM-IV disorders in the US National Comorbidity Survey Replication Adolescent Supplement (NCS-A). Psychol Med 42:1997–2010. 10.1017/S0033291712000025 22273480 10.1017/S0033291712000025 PMC3448706

[CR50] Kranzler HR, Zhou H, Kember RL, Vickers Smith R, Justice AC, Damrauer S, Tsao PS, Klarin D, Baras A, Reid J, Overton J, Rader DJ, Cheng Z, Tate JP, Becker WC, Concato J, Xu K, Polimanti R, Zhao H, Gelernter J (2019) Genome-wide association study of alcohol consumption and use disorder in 274,424 individuals from multiple populations. Nat Commun 2019 10(1 10):1–11. 10.1038/s41467-019-09480-8 10.1038/s41467-019-09480-8 PMC6445072 30940813

[CR51] Lorant V, Deliège D, Eaton W, Robert A, Philippot P, Ansseau M (2003) Socioeconomic inequalities in depression: a meta-analysis. Am J Epidemiol 157:98–112. 10.1093/AJE/KWF182 12522017 10.1093/aje/kwf182

[CR52] Maj M (2005) The aftermath of the concept of psychiatric comorbidity. Psychother Psychosom 74:67–68. 10.1159/000083164 15741755 10.1159/000083164

[CR45] McHugh KR, Weiss RD (2019) Alcohol use disorder and depressive disorders. Alcohol Res 40:e1–e8. 10.35946/ARCR.V40.1.01 10.35946/arcr.v40.1.01 PMC6799954 31649834

[CR53] Meng X, Navoly G, Giannakopoulou O, Levey DF, Koller D et al (2024) Multi-ancestry genome-wide association study of major depression aids locus discovery, fine mapping, gene prioritization and causal inference. Nat Genet 2024 56(2):222–233. 10.1038/s41588-023-01596-4 10.1038/s41588-023-01596-4 PMC10864182 38177345

[CR54] Mitchell BL, Thorp JG, Wu Y, Campos AI, Nyholt DR, Gordon SD, Whiteman DC, Olsen CM, Hickie IB, Martin NG, Medland SE, Wray NR, Byrne EM (2021) Polygenic risk scores derived from varying definitions of depression and risk of depression. JAMA Psychiatry 78:1152–1160. 10.1001/JAMAPSYCHIATRY.2021.1988 34379077 10.1001/jamapsychiatry.2021.1988 PMC8358814

[CR55] Müller M, Ajdacic-Gross V, Vetrella AB, Preisig M, Castelao E, Lasserre A, Rodgers S, Rössler W, Vetter S, Seifritz E, Vandeleur C (2020) Subtypes of alcohol use disorder in the general population: A latent class analysis. Psychiatry Res 285:112712. 10.1016/J.PSYCHRES.2019.112712 31837815 10.1016/j.psychres.2019.112712

[CR56] Murphy A, Schilder B, Skene N (2025) MungeSumstats: Standardise summary statistics from GWAS. R package version 1.16.0. 10.18129/B9.bioc.MungeSumstats

[CR57] Navrady LB, Adams MJ, Chan SWY, Ritchie SJ, Mcintosh AM (2018) Genetic risk of major depressive disorder: the moderating and mediating effects of neuroticism and psychological resilience on clinical and self-reported depression. Psychol Med 48:1890. 10.1017/S0033291717003415 29183409 10.1017/S0033291717003415 PMC6088772

[CR58] Nordgaard J, Nielsen KM, Rasmussen AR, Henriksen MG (2023) Psychiatric comorbidity: a concept in need of a theory. Psychol Med 53:5902. 10.1017/S0033291723001605 37264812 10.1017/S0033291723001605 PMC10520580

[CR60] Palk AC, Dalvie S, De Vries J, Martin AR, Stein DJ (2019) Potential use of clinical polygenic risk scores in psychiatry - ethical implications and communicating high polygenic risk. Philos Ethics Humanit Med. 10.1186/S13010-019-0073-8 30813945 10.1186/s13010-019-0073-8 PMC6391805

[CR61] Peyrot WJ, Lee SH, Milaneschi Y, Abdellaoui A, Byrne EM et al (2015) The association between lower educational attainment and depression owing to shared genetic effects? Results in ~ 25,000 subjects. Mol Psychiatry 20:735. 10.1038/MP.2015.50 25917368 10.1038/mp.2015.50 PMC4610719

[CR62] Prescott CA, Aggen SH, Kendler KS (2000) Sex-specific genetic influences on the comorbidity of alcoholism and major depression in a population-based sample of US twins. Arch Gen Psychiatry 57:803–811. 10.1001/ARCHPSYC.57.8.803 10920470 10.1001/archpsyc.57.8.803

[CR63] Rabinowitz JA, Campos AI, Benjet C, Su J, Macias-Kauffer L, Méndez E, Martinez-Levy GA, Cruz-Fuentes CS, Rentería ME (2020) Depression polygenic scores are associated with major depressive disorder diagnosis and depressive episode in Mexican adolescents. J Affect Disord Rep 2:100028. 10.1016/J.JADR.2020.100028

[CR64] Rehm J, Mathers C, Popova S, Thavorncharoensap M, Teerawattananon Y, Patra J (2009) Global burden of disease and injury and economic cost attributable to alcohol use and alcohol-use disorders. Lancet 373:2223–2233. 10.1016/S0140-6736(09)60746-7 19560604 10.1016/S0140-6736(09)60746-7

[CR65] Ribasés M, Mitjans M, Hartman CA, Soler Artigas M, Demontis D, Larsson H, Ramos-Quiroga JA, Kuntsi J, Faraone SV, Børglum AD, Reif A, Franke B, Cormand B (2023) Genetic architecture of ADHD and overlap with other psychiatric disorders and cognition-related phenotypes. Neurosci Biobehav Rev 153:105313. 10.1016/J.NEUBIOREV.2023.105313 37451654 10.1016/j.neubiorev.2023.105313 PMC10789879

[CR66] Rosoff DB, Clarke TK, Adams MJ, McIntosh AM, Davey Smith G, Jung J, Lohoff FW (2021) Educational attainment impacts drinking behaviors and risk for alcohol dependence: results from a two-sample Mendelian randomization study with ~ 780,000 participants. Mol Psychiatry 26:1119. 10.1038/S41380-019-0535-9 31649322 10.1038/s41380-019-0535-9 PMC7182503

[CR67] Ruan Y, Lin YF, Feng YCA, Chen CY, Lam M et al (2022) Improving polygenic prediction in ancestrally diverse populations. Nat Genet 54:573. 10.1038/S41588-022-01054-7 35513724 10.1038/s41588-022-01054-7 PMC9117455

[CR68] Sanchez-Roige S, Palmer AA, Fontanillas P, Elson SL, Adams MJ et al (2019) Genome-wide association study meta-analysis of the Alcohol Use Disorder Identification Test (AUDIT) in two population-based cohorts. Am J Psychiatry 176:107. 10.1176/APPI.AJP.2018.18040369 30336701 10.1176/appi.ajp.2018.18040369 PMC6365681

[CR69] Sanchez-Villegas A, Schlatter J, Ortuno F, Lahortiga F, Pla J, Benito S, Martinez-Gonzalez MA (2008) Validity of a self-reported diagnosis of depression among participants in a cohort study using the Structured Clinical Interview for DSM-IV (SCID-I). BMC Psychiatry 8:43. 10.1186/1471-244X-8-43 18558014 10.1186/1471-244X-8-43 PMC2447836

[CR70] Seeley JR, Farmer RF, Kosty DB, Gau JM (2019) Prevalence, incidence, recovery, and recurrence of alcohol use disorders from childhood to age 30. Drug Alcohol Depend 194:45–50. 10.1016/J.DRUGALCDEP.2018.09.012 30399499 10.1016/j.drugalcdep.2018.09.012 PMC7018515

[CR71] Sherry ST, Ward MH, Kholodov M, Baker J, Phan L, Smigielski EM, Sirotkin K (2001) DbSNP: The NCBI database of genetic variation. Nucleic Acids Res 29:308–311. 10.1093/nar/29.1.308 11125122 10.1093/nar/29.1.308 PMC29783

[CR72] Shi Y, Sprooten E, Mulders P, Vrijsen J, Bralten J, Demontis D, Børglum AD, Walters GB, Stefansson K, van Eijndhoven P, Tendolkar I, Franke B, Mota NR (2023) Multi-polygenic scores in psychiatry: From disorder specific to transdiagnostic perspectives. Am J Med Genet B Neuropsychiatr Genet 195:e32951. 10.1002/AJMG.B.32951 37334623 10.1002/ajmg.b.32951 PMC10803201

[CR73] Sivakumaran S, Agakov F, Theodoratou E, Prendergast JG, Zgaga L, Manolio T, Rudan I, McKeigue P, Wilson JF, Campbell H (2011) Abundant pleiotropy in human complex diseases and traits. Am J Hum Genet 89:607. 10.1016/J.AJHG.2011.10.004 22077970 10.1016/j.ajhg.2011.10.004 PMC3213397

[CR59] Østergaard SD, Jensen SOW, Bech P (2011) The heterogeneity of the depressive syndrome: when numbers get serious. Acta Psychiatr Scand 124:495–496. 10.1111/J.1600-0447.2011.01744.X 21838736 10.1111/j.1600-0447.2011.01744.x

[CR74] Strom NI, Grove J, Meier SM, Bækvad-Hansen M, Becker Nissen J, Damm Als T, Halvorsen M, Nordentoft M, Mortensen PB, Hougaard DM, Werge T, Mors O, Børglum AD, Crowley JJ, Bybjerg-Grauholm J, Mattheisen M (2021) Polygenic heterogeneity across obsessive-compulsive disorder subgroups defined by a comorbid diagnosis. Front Genet 12:711624. 10.3389/FGENE.2021.711624/FULL 34531895 10.3389/fgene.2021.711624 PMC8438210

[CR75] Stuart AL, Pasco JA, Jacka FN, Brennan SL, Berk M, Williams LJ (2014) Comparison of self-report and structured clinical interview in the identification of depression. Compr Psychiatry 55:866–869. 10.1016/J.COMPPSYCH.2013.12.019 24467941 10.1016/j.comppsych.2013.12.019

[CR14] Substance Abuse and Mental Health Services Administration (2023) Key substance use and mental health indicators in the United States: Results from the 2022 National Survey on Drug Use and Health (HHS Publication No. PEP23-07-01-006, NSDUH Series H-58). Center for Behavioral Health Statistics and Quality, Substance Abuse and Mental Health Services Administration

[CR76] Sullivan PF, Neale MC, Kendler KS (2000) Genetic epidemiology of major depression: review and meta-analysis. Am J Psychiatry 157:1552–1562. 10.1176/APPI.AJP.157.10.1552 11007705 10.1176/appi.ajp.157.10.1552

[CR77] Tabrizi F, Rosén J, Grönvall H, William-Olsson VR, Arner E, Magnusson PKE, Palm C, Larsson H, Viktorin A, Bernhardsson J, Björkdahl J, Jansson B, Sundin Ö, Zhou X, Speed D, Åhs F (2025) Heritability and polygenic load for comorbid anxiety and depression. Transl Psychiatry 2025 15(1 15):1–10. 10.1038/s41398-025-03325-3 10.1038/s41398-025-03325-3 PMC11947153 40140358

[CR78] Takahashi T, Lapham G, Chavez LJ, Lee AK, Williams EC, Richards JE, Greenberg D, Rubinsky A, Berger D, Hawkins EJ, Merrill JO, Bradley KA (2017) Comparison of DSM-IV and DSM-5 criteria for alcohol use disorders in VA primary care patients with frequent heavy drinking enrolled in a trial. Addict Sci Clin Pract 12:1–10. 10.1186/S13722-017-0082-0/TABLES/3 28716049 10.1186/s13722-017-0082-0 PMC5514480

[CR79] Tang L, Tang R, Zheng J, Zhao P, Zhu R, Tang Y, Zhang X, Gong X, Wang F (2025) Dissecting biological heterogeneity in major depressive disorder based on neuroimaging subtypes with multi-omics data. Transl Psychiatry 15(1):72. 10.1038/s41398-025-03286-7 10.1038/s41398-025-03286-7 PMC11876359 40032862

[CR80] Torvik FA, Ystrom E, Røysamb E, Czajkowski N, Knudsen GP, Rosenström TH, Tambs K, Gillespie N, Kendler KS, Reichborn-Kjennerud T (2017) Stability and change in Etiological factors for Alcohol use disorder and major depression. J Abnorm Psychol 126:812–822. 10.1037/ABN0000280 28541064 10.1037/abn0000280 PMC5546937

[CR81] Tozzi L, Zhang X, Pines A, Olmsted AM, Zhai ES, Anene ET, Chesnut M, Holt-Gosselin B, Chang S, Stetz PC, Ramirez CA, Hack LM, Korgaonkar MS, Wintermark M, Gotlib IH, Ma J, Williams LM (2024) Personalized brain circuit scores identify clinically distinct biotypes in depression and anxiety. Nat Med 2024 30:7302076–7302087. 10.1038/s41591-024-03057-9 10.1038/s41591-024-03057-9 PMC11271415 38886626

[CR82] Valderas JM, Starfield B, Sibbald B, Salisbury C, Roland M (2009) Defining comorbidity: implications for understanding health and health services. Ann Fam Med 7:357. 10.1370/AFM.983 19597174 10.1370/afm.983 PMC2713155

[CR83] van Buuren S, Groothuis-Oudshoorn K (2011) mice: multivariate imputation by chained equations in R. J Stat Softw 45:1–67. 10.18637/JSS.V045.I03

[CR84] Verhulst B, Neale MC, Kendler KS (2015) The heritability of alcohol use disorders: a meta-analysis of twin and adoption studies. Psychol Med 45:1061. 10.1017/S0033291714002165 25171596 10.1017/S0033291714002165 PMC4345133

[CR85] Villarroel MA, Terlizzi EP (2020) Symptoms of depression among adults: United States, 2019. NCHS Data Brief, no 379. National Center for Health Statistics, Hyattsville 33054920

[CR86] Walters RK, Polimanti R, Johnson EC, McClintick JN, Adams MJ et al (2018) Trans-ancestral GWAS of alcohol dependence reveals common genetic underpinnings with psychiatric disorders. Nat Neurosci 21:1656. 10.1038/S41593-018-0275-1 30482948 10.1038/s41593-018-0275-1 PMC6430207

[CR87] Wang Y, Kanai M, Tan T, Kamariza M, Tsuo K, Yuan K, Zhou W, Okada Y, Huang H, Turley P, Atkinson EG, Martin AR (2023) Polygenic prediction across populations is influenced by ancestry, genetic architecture, and methodology. Cell Genomics. 10.1016/J.XGEN.2023.100408 37868036 10.1016/j.xgen.2023.100408 PMC10589629

[CR88] Wendt FR, Pathak GA, Tylee DS, Goswami A, Polimanti R (2020) Heterogeneity and polygenicity in psychiatric disorders: a genome-wide perspective. Chronic Stress 4:2470547020924844. 10.1177/2470547020924844 32518889 10.1177/2470547020924844 PMC7254587

[CR89] Wray NR, Ripke S, Mattheisen M, Trzaskowski M, Byrne EM et al (2018) Genome-wide association analyses identify 44 risk variants and refine the genetic architecture of major depression. Nat Genet 2018 50(5):668–681. 10.1038/s41588-018-0090-3 10.1038/s41588-018-0090-3 PMC5934326 29700475

[CR90] Yoon G, Petrakis IL, Rosenheck RA (2015) Correlates of major depressive disorder with and without comorbid alcohol use disorder nationally in the veterans health administration. Am J Addict 24:419–426. 10.1111/AJAD.12219 25950244 10.1111/ajad.12219

[CR91] Zare H, Meyerson NS, Nwankwo CA, Thorpe RJ (2022) How income and income inequality drive depressive symptoms in U.S. adults, does sex matter: 2005–2016. Int J Environ Res Public Health 19:2005–2016. 10.3390/IJERPH19106227 35627767 10.3390/ijerph19106227 PMC9140340

[CR93] Zhou H, Sealock JM, Sanchez-Roige S, Clarke TK, Levey DF, Cheng Z, Li B, Polimanti R, Kember RL, Smith RV, Thygesen JH, Morgan MY, Atkinson SR, Thursz MR, Nyegaard M, Mattheisen M, Børglum AD, Johnson EC, Justice AC, Palmer AA, McQuillin A, Davis LK, Edenberg HJ, Agrawal A, Kranzler HR, Gelernter J (2020) Genome-wide meta-analysis of problematic alcohol use in 435,563 individuals yields insights into biology and relationships with other traits. Nat Neurosci 23:809–818. 10.1038/S41593-020-0643-5 32451486 10.1038/s41593-020-0643-5 PMC7485556

[CR92] Zhou H, Kember RL, Deak JD, Xu H, Toikumo S et al (2023) Multi-ancestry study of the genetics of problematic alcohol use in over 1 million individuals. Nat Med 2023 29(12):3184–3192. 10.1038/s41591-023-02653-5 10.1038/s41591-023-02653-5 PMC10719093 38062264

[CR94] Zhu T, Becquey C, Chen Y, Lejuez CW, Li CSR, Bi J (2022) Identifying alcohol misuse biotypes from neural connectivity markers and concurrent genetic associations. Transl Psychiatry 12:253. 10.1038/s41398-022-01983-1 35710901 10.1038/s41398-022-01983-1 PMC9203552

