# Supplementary material for: The Contributions of Multiple Polygenic Scores in Predicting Liability for Major Depressive Disorder and Its Comorbidity with Alcohol Use Disorder
Source: Behav Genet. 2026 Apr 10;56(2):80–98. doi: 10.1007/s10519-026-10263-3 (PMC13132973; doi:10.1007/s10519-026-10263-3)
Supplement: Supplementary file 1 — Supplementary Material 1 [file 10519_2026_10263_MOESM1_ESM.docx]

Supplemental File

TITLE: The Contributions of Multiple Polygenic Scores in Predicting Liability for Major Depressive Disorder and its Comorbidity with Alcohol Use Disorder

Jonathan L. Wells, MHS^1,2^, Jill A. Rabinowitz, PhD^3^, Brion S. Maher, PhD^4^, Amanda Elswick Gentry^2,5^, PhD^2^, James B. Burch, PhD^1^, Elizabeth C. Prom-Wormley, MPH, PhD^1,2^

1. Department of Epidemiology, Virginia Commonwealth University School of Public Health, Richmond, VA 23219, USA.
2. Virginia Institute for Psychiatric and Behavioral Genetics, Virginia Commonwealth University, Richmond, VA 23298, USA.
3. Department of Psychiatry, Robert Wood Johnson Medical School, Rutgers University, New Brunswick, NJ 08901, USA.
4. Department of Mental Health, Bloomberg School of Public Health, Johns Hopkins University, Baltimore, MD 21205, USA.
5. BeOne Medicines Ltd., Basel, Switzerland.

Corresponding author:

Jonathan L. Wells

830 E Main St. Floor 8

Richmond, VA 23219

[wellsjl4@vcu.edu](mailto:wellsjl4@vcu.edu)

| **Supplemental Table 1. Unrelated and Related Sample Between Group Differences** | | | |
| --- | --- | --- | --- |
| **Variable** | **Unrelated** | **Related** | **p-value** |
|  | **N (%)** | **N (%)** | **N (%)** |
| **Race/Ethnicity** | |  | <0.001 |
| Asian | 26 (0.4) | 1 (0.1) |  |
| Black | 1,673 (23.0) | 266 (18.8) |  |
| Hispanic | 813 (11.2) | 131 (9.3) |  |
| Native American | 20 (0.3) | 2 (0.1) |  |
| White | 4,741 (65.1) | 1,013 (71.6) |  |
| **Biological Sex** | |  | 0.145 |
| Female | 3,889 (53.4) | 725 (51.3) |  |
| Male | 3,390 (46.6) | 689 (48.7) |  |
| **Mean Adolescent HHI (SD)** | 4.45 (1.98) | 4.49 (2.03) | 0.571 |
| **Mean Adolescent HHI (SD)** | 5.15 (2.06) | 5.33 (2.01) | 0.004 |
| **Educational Attainment** | | | 0.013 |
| Did not complete high school | 608 (8.4) | 135 (9.5) |  |
| High school | 1250 (17.2) | 234 (16.5) |  |
| Some college/vocational/  technical training | 2878 (39.5) | 499 (35.3) |  |
| Completed college/vocational/  technical training | 1747 (24.0) | 382 (27.0) |  |
| Graduate degree | 796 (10.9) | 164 (11.6) |  |
| **Mean Age at Wave 4 (SD)** | 28.48 (1.79) | 28.44 (1.71) | 0.523 |
| **MDD** |  |  | 0.270 |
| Controls | 4075 (56.0) | 778 (55.0) |  |
| Cases | 735 (10.1) | 163 (11.5) |  |
| Excluded | 2469 (33.9) | 473 (33.5) |  |
| **AUD** |  |  | 0.590 |
| Controls | 4075 (56.0) | 778 (55.0) |  |
| Cases | 1962 (27.0) | 379 (26.8) |  |
| Excluded | 1242 (17.1) | 257 (18.2) |  |
| **Comorbid MDD-AUD** |  |  | 0.641 |
| Controls | 4075 (56.0) | 778 (55.0) |  |
| Cases | 507 (7.0) | 94 (6.6) |  |
| Excluded | 2697 (37.1) | 542 (38.3) |  |
| **META-MDD PGS** | 0.01 (1.00) | 0.01 (1.04) | 0.95 |
| **META-AUD PGS** | 0.00 (1.00) | -0.04 (1.00) | 0.206 |
| **Total Sample Size** | **7279** | **1414** |  |
| HHI = Household Income MDD = Major Depressive Disorder, AUD = Alcohol Use Disorder, PGS = polygenic score; Significance was determined with a p < 0.05 | | | |

| **Supplement Table 2. DSM-IV Alcohol Dependence and Alcohol Abuse Questions** | |
| --- | --- |
| **Question** | **Coding** |
| **Alcohol Dependence** | |
| 1. Have you ever found that you had to drink more than you used to in order to get the effect you wanted? | Yes = 1; No = 0 |
| 2. Has there ever been a period when you spent a lot of time using, acquiring it, or getting over the effect of alcohol? | Yes = 1; No = 0 |
| 3. Have you often drunk more alcohol or drank alcohol longer than you intended? | Yes = 1; No = 0 |
| 4. Has there ever been a period of time when you wanted to quit or cut down on your use of alcohol? | Yes = 1; No = 0 |
| 5. During the first few hours of not using alcohol, do you experience withdrawal symptoms such as craving, feeling depressed, anxious, restless or irritable, having trouble concentrating, feeling tired or weak, having trouble sleeping, or a change in appetite? | Yes = 1; No = 0 |
| 6. Have you ever continue to use alcohol after you realized using alcohol was causing you any emotional problems (such as feeling depressed or empty, feeling irritable or aggressive, feeling paranoid or confused, feeling anxious or tense, being jumpy or easily startled) or causing you any health problems (such as a persistent cough, sore throat or sinus problems, heart pounding, headaches or dizziness, or sexual difficulties)? | Yes = 1; No = 0 |
| 7. Have you ever given up or cut down on important activities that would interfere with your alcohol use like getting together with friends or relatives, going to work or school, participating in sports, or anything else? | Yes = 1; No = 0 |
| **Alcohol Abuse (**How many times has each of the following things ever happened?) | |
| 1. How often has your alcohol use interfered with your responsibilities at work or school? | More than one time = 1; Else = 0 |
| 2. How often have you been under the influence of alcohol when you could have gotten yourself or others hurt, or put yourself or others at risk, including unprotected sex? | More than one time = 1; Else = 0 |
| 3. How often have you had legal problems because of your alcohol use, like being arrested for disturbing the peace or anything else? | More than one time = 1; Else = 0 |
| 4. How often have you had problems with your family, friends, or people at work or school because of your alcohol use? | More than one time = 1; Else = 0 |

**Supplemental Figure 1.** Normalized META-MDD PGS Scores by Outcome Group


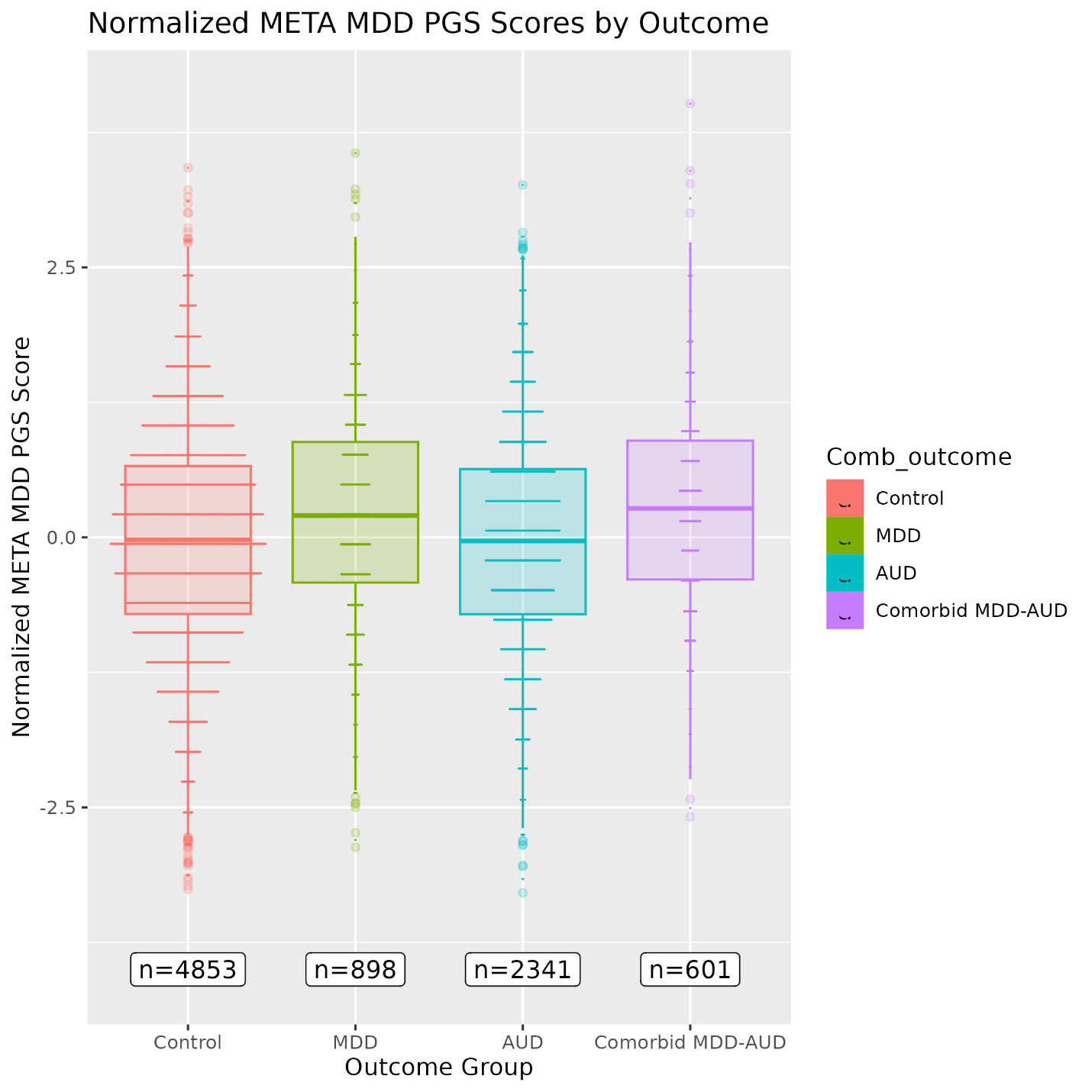


**Supplemental Figure 2.** Normalized META-AUD PGS Scores by Outcome Group


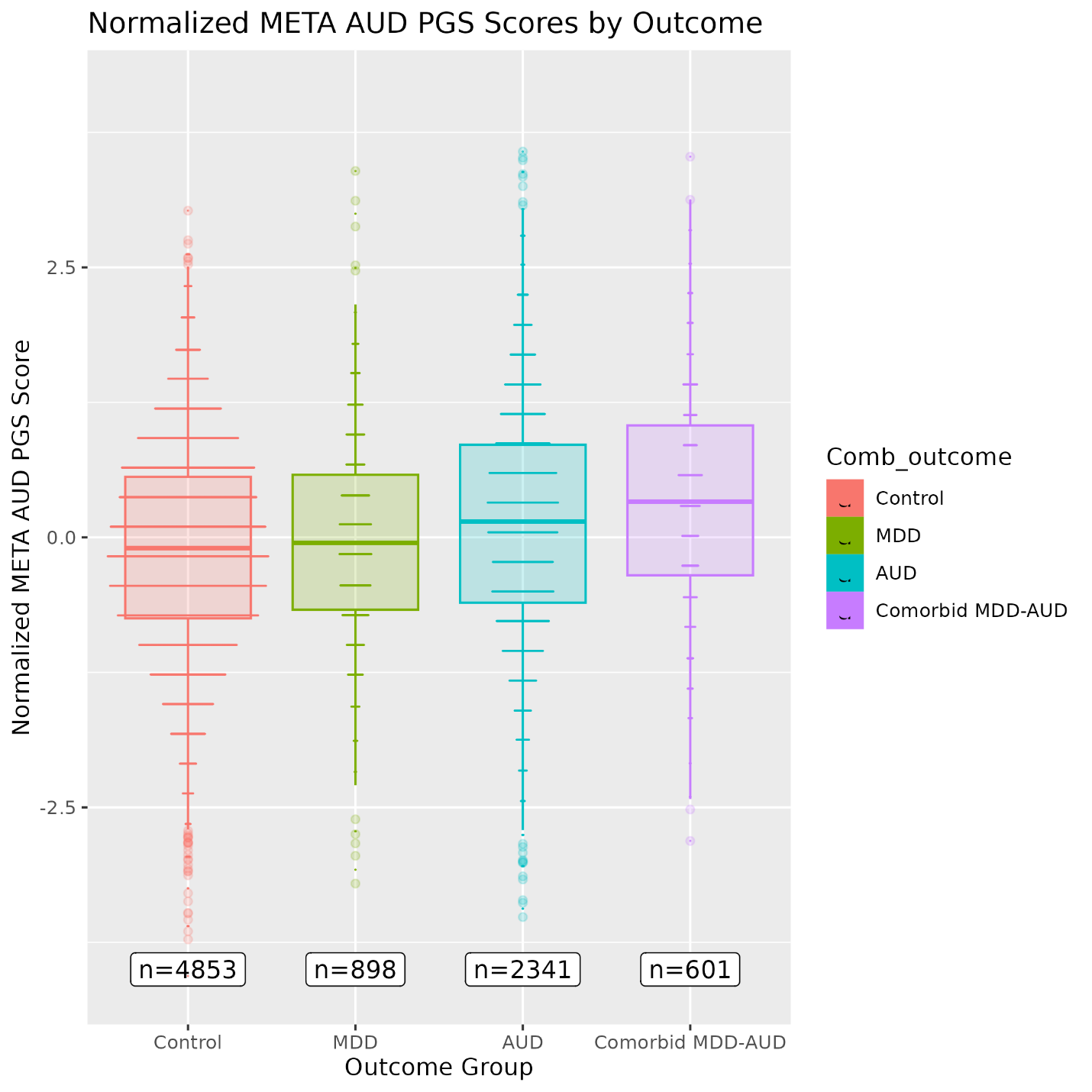


| **Supplemental Table 3. Outcome Distribution by Ancestry in Add Health (Non-Exclusion Case Groups)** | | | |
| --- | --- | --- | --- |
| **Case Status** | **European** | **African** | **Hispanic** |
| MDD | 1051 (20.1%) | 205 (11.2%) | 110 (12.1%) |
| Control | 4167 (79.9%) | 1631 (88.8%) | 801 (87.9%) |
| AUD | 2106 (40.4%) | 306 (16.7%) | 277 (30.4%) |
| Control | 3112 (59.6%) | 1530 (83.3%) | 634 (69.6%) |
| Comorbid MDD-AUD | 461 (8.8%) | 43 (2.3%) | 40 (4.4%) |
| Control | 4757 (91.2%) | 1793 (97.7%) | 871 (95.6%) |
| **Total** | **5218 (100%)** | **1836 (100%)** | **911 (100%)** |
|  | | | |

| **Supplemental Table 4**. EUR PGS and OLD PGS Model Estimates and Performance in EUR Ancestry Population | | | | | | | |
| --- | --- | --- | --- | --- | --- | --- | --- |
| **Model Name** | **McFadden Adjusted R^2^** | **AIC** | **BIC** | **MDD PGS OR (95% CI)** | **MDD PGS p-value** | **AUD PGS OR (95% CI)** | **AUD PGS p-value** |
| MDD Liability Models | | | | | | | |
| Base M0 | 4.40% | 2889.6 | 3016.5 | — | — | — | — |
| EUR PGS M1 | 5.36% | 2860.6 | 2993.5 | 1.30 (1.21-1.40) | 3.86E-08 | — | — |
| EUR PGS M2 | 4.64% | 2882.3 | 3015.3 | — | — | 1.18 (1.07-1.29) | 0.002 |
| **EUR PGS M3** | **5.46%** | **2857.6** | **2996.6** | **1.28 (1.19-1.38)** | **3.10E-07** | **1.13 (1.02-1.24)** | **0.025** |
| OLD PGS M1 | 5.20% | 2865.3 | 2998.3 | 1.28 (1.18-1.37) | 4.15E-07 | — | — |
| OLD PGS M2 | 4.35% | 2891.0 | 3024.0 | — | — | 0.97 (0.87-1.06) | 0.452 |
| OLD PGS M3 | 5.16% | 2866.6 | 3005.6 | 1.28 (1.18-1.38) | 3.76E-07 | 0.96 (0.87-1.05) | 0.382 |
| AUD Liability Models | | | | | | | |
| Base M0 | 1.95% | 5479.7 | 5612.8 | — | — | — | — |
| EUR PGS M1 | 1.95% | 5479.8 | 5619.2 | 1.05 (0.98-1.11) | 0.170 | — | — |
| **EUR PGS M2** | **5.35%** | **5289.7** | **5429.1** | **—** | **—** | **1.61 (1.54-1.68)** | **4.84E-40** |
| EUR PGS M3 | 5.33% | 5291.2 | 5436.9 | 0.98 (0.91-1.05) | 0.494 | 1.62 (1.54-1.69) | 9.18E-40 |
| OLD PGS M1 | 1.95% | 5479.7 | 5619.0 | 1.05 (0.98-1.11) | 0.150 | — | — |
| OLD PGS M2 | 1.92% | 5481.7 | 5621.1 | — | — | 1.00 (0.93-1.06) | 0.994 |
| OLD PGS M3 | 1.92% | 5481.7 | 5627.4 | 1.05 (0.98-1.11) | 0.150 | 1.00 (0.93-1.06) | 0.986 |
| Comorbid MDD-AUD Liability Models | | | | | | | |
| Base M0 | 2.71% | 2495.1 | 2621.1 | — | — | — | — |
| EUR PGS M1 | 4.29% | 2454.7 | 2586.7 | 1.41 (1.30-1.51) | 1.63E-10 | — | — |
| EUR PGS M2 | 9.29% | 2326.4 | 2458.4 | — | — | 2.11 (1.99-2.23) | 1.86E-34 |
| **EUR PGS M3** | **10.03%** | **2307.5** | **2445.5** | **1.29 (1.18-1.40)** | **5.97E-06** | **2.04 (1.91-2.16)** | **8.89E-31** |
| OLD PGS M1 | 3.77% | 2468.1 | 2600.1 | 1.33 (1.22-1.43) | 1.11E-07 | — | — |
| OLD PGS M2 | 2.68% | 2496.0 | 2628.0 | — | — | 0.95 (0.84-1.05) | 0.302 |
| OLD PGS M3 | 3.74% | 2468.7 | 2606.7 | 1.33 (1.22-1.44) | 9.45E-08 | 0.94 (0.83-1.04) | 0.237 |
| The best performing model has been bolded for each outcome. EUR = European Ancestry, PGS = Polygenic Score, META = Meta-analyzed PRS-CSx PGS, OLD = Previously Generated Scores in Add Health, AIC = Akaike Information Criterion, BIC = Bayesian Information Criterion, OR = Odds Ratio, MDD = Major Depressive Disorder, AUD = Alcohol Use Disorder, M0 = Covariates Only, M1 = MDD PGS + Covariates, M2 = AUD PGS + Covariates, M3 = MDD PGS + AUD PGS + Covariates | | | | | | | |

| **Supplemental Table 5**. AFR PGS Model Estimates and Performance in AFR Ancestry Population | | | | | | | |
| --- | --- | --- | --- | --- | --- | --- | --- |
| **Model Name** | **McFadden Adjusted R^2^** | **AIC** | **BIC** | **MDD PGS OR (95% CI)** | **MDD PGS p-value** | **AUD PGS OR (95% CI)** | **AUD PGS p-value** |
| MDD Liability Models | | | | | | | |
| **Base M0** | **0.49%** | **1028.4** | **1151.1** | **—** | **—** | **—** | **—** |
| AFR PGS M1 | 0.35% | 1029.9 | 1157.9 | 1.06 (0.89-1.24) | 0.480 | — | — |
| AFR PGS M2 | 0.34% | 1030.0 | 1158.0 | — | — | 0.94 (0.77-1.12) | 0.512 |
| AFR PGS M3 | 0.21% | 1031.3 | 1164.6 | 1.07 (0.90-1.25) | 0.416 | 0.93 (0.76-1.11) | 0.441 |
| AUD Liability Models | | | | | | | |
| **Base M0** | **0.95%** | **1426.9** | **1551.0** | **—** | **—** | **—** | **—** |
| AFR PGS M1 | 0.81% | 1428.9 | 1558.4 | 0.99 (0.84-1.13) | 0.838 | — | — |
| AFR PGS M2 | 0.84% | 1428.5 | 1558.0 | — | — | 1.05 (0.91-1.19) | 0.504 |
| AFR PGS M3 | 0.71% | 1430.4 | 1565.3 | 0.98 (0.84-1.12) | 0.775 | 1.05 (0.91-1.19) | 0.485 |
| Comorbid MDD-AUD Liability Models | | | | | | | |
| **Base M0** | **-4.65%** | **402.7** | **523.5** | **—** | **—** | **—** | **—** |
| AFR PGS M1 | -5.15% | 404.7 | 530.7 | 1.04 (0.71-1.37) | 0.810 | — | — |
| AFR PGS M2 | -5.17% | 404.7 | 530.7 | — | — | 0.99 (0.66-1.32) | 0.948 |
| AFR PGS M3 | -5.67% | 406.6 | 537.9 | 1.04 (0.71-1.37) | 0.803 | 0.98 (0.65-1.32) | 0.926 |
| The best performing model has been bolded for each outcome. AFR = African Ancestry, PGS = Polygenic Score, AIC = Akaike Information Criterion, BIC = Bayesian Information Criterion, OR = Odds Ratio, MDD = Major Depressive Disorder, AUD = Alcohol Use Disorder, M0 = Covariates Only, M1 = MDD PGS + Covariates, M2 = AUD PGS + Covariates, M3 = MDD PGS + AUD PGS + Covariates | | | | | | | |

| **Supplemental Table 6**. AMR PGS Model Estimates and Performance in AMR Ancestry Population | | | | | | | |
| --- | --- | --- | --- | --- | --- | --- | --- |
| **Model Name** | **McFadden Adjusted R^2^** | **AIC** | **BIC** | **MDD PGS OR (95% CI)** | **MDD PGS p-value** | **AUD PGS OR (95% CI)** | **AUD PGS p-value** |
| MDD Liability Models | | | | | | | |
| Base M0 | -1.28% | 446.1 | 548.5 | — | — | — | — |
| AMR PGS M1 | -1.65% | 447.7 | 554.6 | 1.09 (0.81-1.38) | 0.534 | — | — |
| **AMR PGS M2** | **-0.93%** | **444.6** | **551.4** | **—** | **—** | **1.30 (1.02-1.57)** | **0.062** |
| AMR PGS M3 | -1.30% | 446.2 | 557.5 | 1.09 (0.81-1.38) | 0.541 | 1.30 (1.02-1.57) | 0.062 |
| AUD Liability Models | | | | | | | |
| **Base M0** | **3.58%** | **938.1** | **1045.9** | **—** | **—** | **—** | **—** |
| AMR PGS M1 | 3.55% | 938.4 | 1050.9 | 0.89 (0.72-1.07) | 0.192 | — | — |
| AMR PGS M2 | 3.49% | 939.0 | 1051.5 | — | — | 0.92 (0.75-1.08) | 0.295 |
| AMR PGS M3 | 3.46% | 939.3 | 1056.4 | 0.89 (0.72-1.07) | 0.189 | 0.91 (0.75-1.08) | 0.290 |
| Comorbid MDD-AUD Liability Models | | | | | | | |
| Base M0 | 2.00% | 288.6 | 389.9 | — | — | — | — |
| AMR PGS M1 | 1.71% | 289.4 | 395.1 | 1.24 (0.83-1.65) | 0.287 | — | — |
| **AMR PGS M2** | **2.41%** | **287.4** | **393.0** | **—** | **—** | **1.40 (1.02-1.78)** | **0.076** |
| AMR PGS M3 | 2.12% | 288.2 | 398.3 | 1.24 (0.83-1.65) | 0.289 | 1.41 (1.02-1.79) | 0.077 |
| The best performing model has been bolded for each outcome. AMR = Admixed American Ancestry, PGS = Polygenic Score, AIC = Akaike Information Criterion, BIC = Bayesian Information Criterion, OR = Odds Ratio, MDD = Major Depressive Disorder, AUD = Alcohol Use Disorder, M0 = Covariates Only, M1 = MDD PGS + Covariates, M2 = AUD PGS + Covariates, M3 = MDD PGS + AUD PGS + Covariates | | | | | | | |

| **Supplemental Table 7**. META PGS Model Estimates and Performance for Non-Excluded Case Definitions | | | | | | | |
| --- | --- | --- | --- | --- | --- | --- | --- |
| **Model Name** | **McFadden Adjusted R^2^** | **AIC** | **BIC** | **MDD PGS OR (95% CI)** | **MDD PGS p-value** | **AUD PGS OR (95% CI)** | **AUD PGS p-value** |
| MDD Liability Models | | | | | | | |
| Base M0 | 5.82% | 6871.2 | 7031.8 | — | — | — | — |
| META PGS M1 | 6.72% | 6805.2 | 6972.8 | 1.29 (1.23-1.35) | 3.52E-16 | — | — |
| META PGS M2 | 6.11% | 6850.0 | 7017.6 | — | — | 1.16 (1.10-1.22) | 1.56E-06 |
| **META PGS M3** | **6.89%** | **6792.8** | **6967.3** | **1.27 (1.21-1.33)** | **2.60E-14** | **1.13 (1.06-1.19)** | **1.54E-04** |
| AUD Liability Models | | | | | | | |
| Base M0 | 5.64% | 9606.7 | 9767.3 | — | — | — | — |
| META PGS M1 | 5.64% | 9607.2 | 9774.7 | 1.03 (0.98-1.08) | 0.212 | — | — |
| **META PGS M2** | **7.53%** | **9414.8** | **9582.4** | **—** | **—** | **1.42 (1.37-1.47)** | **6.88E-42** |
| META PGS M3 | 7.51% | 9416.5 | 9591.0 | 0.99 (0.93-1.04) | 0.550 | 1.42 (1.37-1.48) | 1.15E-41 |
| Comorbid MDD-AUD Liability Models | | | | | | | |
| Base M0 | 5.19% | 3758.8 | 3919.4 | — | — | — | — |
| META PGS M1 | 6.06% | 3724.1 | 3891.7 | 1.31 (1.22-1.41) | 1.93E-09 | — | — |
| META PGS M2 | 7.44% | 3669.4 | 3837.0 | — | — | 1.54 (1.45-1.63) | 4.00E-21 |
| **META PGS M3** | **7.98%** | **3647.9** | **3822.5** | **1.25 (1.16-1.34)** | **1.46E-06** | **1.50 (1.40-1.59)** | **2.28E-18** |
| The best performing model has been bolded for each outcome. PGS = Polygenic Score, META = Meta-analyzed PRS-CSx PGS, OLD = Previously Generated Scores in Add Health, AIC = Akaike Information Criterion, BIC = Bayesian Information Criterion, OR = Odds Ratio, MDD = Major Depressive Disorder, AUD = Alcohol Use Disorder, M0 = Covariates Only, M1 = MDD PGS + Covariates, M2 = AUD PGS + Covariates, M3 = MDD PGS + AUD PGS + Covariates | | | | | | | |
